# Ion‐Pairing Assemblies of Polarized Charged Porphyrin

**DOI:** 10.1002/chem.202502225

**Published:** 2025-08-07

**Authors:** Ryo Kitayama, Yohei Haketa, Yuto Maruyama, Masaki Fujita, Hiroki Tanaka, Hiroki Horita, Hiromitsu Maeda

**Affiliations:** ^1^ Department of Applied Chemistry College of Life Sciences Ritsumeikan University Kusatsu 525–8577 Japan

**Keywords:** π‐electronic systems, charge‐segregated assemblies, dipole, ion pairs, ion‐pairing assemblies

## Abstract

A porphyrin Au^III^ complex comprising 5,10‐penatafluorophenyl units was synthesized as a dipolar π‐electronic cation. The π‐electronic cation was combined with anions, including a bulky borate anion and planar π‐electronic anions. The dipolar π‐electronic cation formed crystal‐state ion‐pairing assemblies based on antiparallel stacking structures, which were stabilized by interionic dipole–dipole interactions.

## Graphical abstract

The ion pairs, consisting of the polarized porphyrin Au^III^ complex as a dipolar π‐electronic cation, formed crystal‐state ion‐pairing assemblies based on antiparallel stacking structures stabilized by dispersion forces along with reduced electrostatic repulsion originating from the dipole–dipole interactions.

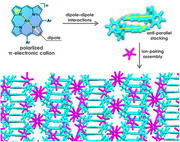

## 1 Introduction

**Figure 1:**
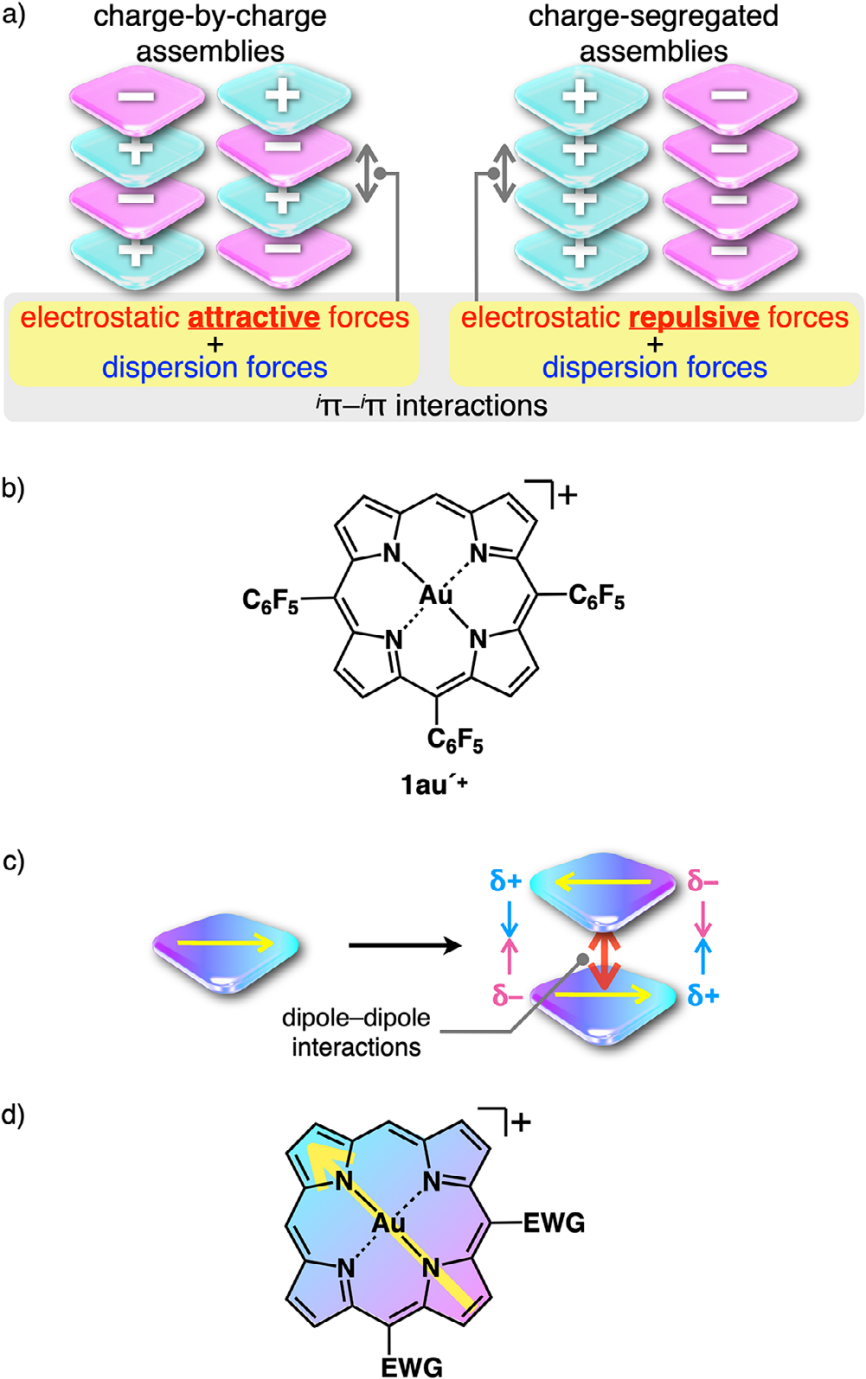
a) Assemblies of charged π‐electronic systems: charge‐by‐charge (left) and charge‐segregated assemblies (right), b) 5,10,15‐tris(pentafluorophenyl)‐substituted porphyrin Au^III^ complex **1au´** ^+^, c) stacking of dipolar π‐electronic cations in antiparallel fashion without the representation of the plus signs on the models, and d) porphyrin Au^III^ complex with electron‐withdrawing groups (EWG) at the 5,10‐positions with the dipole moment indicated by the yellow arrow.

The arrangement of π‐electronic systems is essential for controlling the properties of their assembly states.^[^ ^1^ ^]^ Charged π‐electronic systems are fascinating as building units of assemblies whose assembly modes depend on ^*i*^π–^*i*^π interactions with the contributions from electrostatic and dispersion forces (Figure 1a).^[^ ^2^ ^]^ The ^*i*^π–^*i*^π interactions occur between stacked charged π‐electronic systems in both oppositely and identically charged π‐electronic systems. Charge‐by‐charge and charge‐segregated assemblies formed via ^*i*^π–^*i*^π interactions would provide ferroelectric and electric conductive materials, respectively. The combination of oppositely charged π‐electronic systems is also key to controlling ion‐pairing assembly modes. Recently, porphyrin Au^III^ complexes (e.g., **1au´** ^+^, Figure 1b) were used to form various assemblies in solution and also in the single‐crystal and liquid‐crystal states owing to their positively charged π‐electronic systems.^[^ ^3^ ^]^ Porphyrin Au^III^ complexes have large π‐planes and their electronic states and assembly modes can be modulated by peripheral substituents. Focusing on the assembly modes, in contrast to oppositely charged π‐electronic systems that form charge‐by‐charge stacked structures supported by electrostatic attractive forces (Figure 1a left), identically charged π‐electronic systems are influenced by electrostatic repulsion, ensuring difficulty in their stacking (Figure 1a right).^[^ ^2^ ^]^ Thus, the enhancement of dispersion forces^[^ ^3e^ ^]^ and reduction of electrostatic repulsive forces lead to more attractive ^*i*^π–^*i*^π interactions in charge‐segregated assemblies. Introducing a dipole in charged π‐electronic systems is an efficient way to decrease electrostatic repulsion by dipole–dipole interactions (Figure 1c).^[^ ^4^, ^5^, ^6^ ^]^ In fact, a polarized polymethine‐based cation formed crystal‐state charge‐segregated assemblies that exhibited electric conductivity.^[^ ^5c,e^ ^]^ This strategy to decrease electrostatic repulsion can be applied to dipolar charged porphyrins comprising electron‐withdrawing groups at appropriate positions (Figure 1d). In this study, a porphyrin Au^III^ complex with polarized electronic structure was synthesized to form charge‐segregated assemblies.

## 2 Results and Discussion

**Figure 2:**
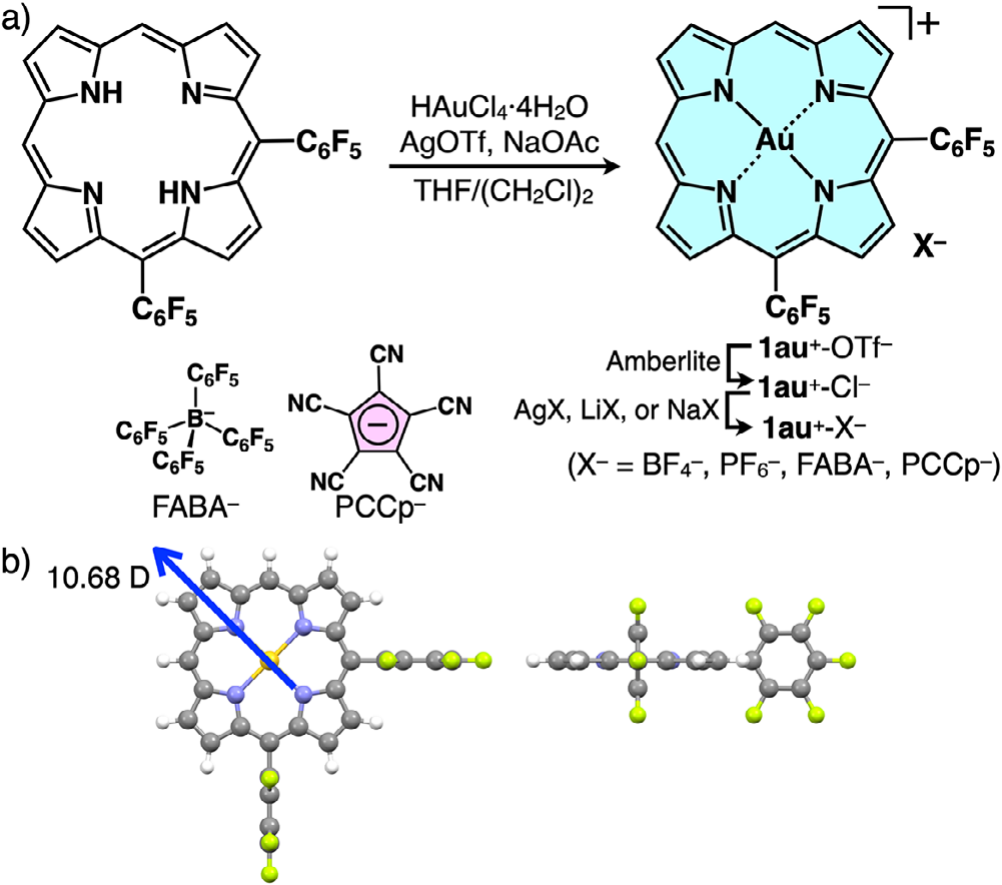
a) Synthesis of 5,10‐bis(pentafluorophenyl)‐substituted porphyrin Au^III^ complex as ion pairs **1au** ^+^‐X^−^ (X^−^ = OTf^−^, Cl^−^, BF_4_^−^, PF_6_^−^, FABA^−^, and PCCp^−^) and b) top and side views of the optimized structure of **1au** ^+^ at B3LYP/6–31+G(d,p) with dipole moment indicated by a blue arrow.

Au^III^ complex of 5,10‐bis(pentafluorophenyl)‐substituted porphyrin **1au** ^+^ was synthesized as an OTf^−^ ion pair in 49% yield by treating the porphyrin prepared according to a previously reported procedure^[^ ^7^ ^]^ that includes using HAuCl_4_·4H_2_O in the presence of AgOTf and NaOAc (Figure 2a). The obtained OTf^‐^ ion pair **1au** ^+^‐OTf^‐^ was converted to the Cl^‐^ ion pair **1au** ^+^‐Cl^‐^ upon treatment with the ion‐exchange resin (Amberlite IRA402BL Cl). **1au** ^+^‐Cl^‐^ was further transformed to the ion pairs with BF_4_^‐^, PF_6_^‐^, B(C_6_F_5_)_4_^‐^ (FABA^‐^), and pentacyanocyclopentadienide (PCCp^‐^) through the ion‐pair metathesis with AgBF_4_, AgPF_6_, LiFABA, and NaPCCp,^[^ ^8^ ^]^ respectively. The produced **1au** ^+^ ion pairs were characterized by ^1^H, ^13^C, and ^19^F NMR (Figures S1–S6,S26,S27) along with ESI‐TOF‐MS. The optimized structure of **1au** ^+^ at B3LYP/6–31+G(d,p) suggested a planar geometry for the porphyrin core unit with a mean‐plane deviation of 0.00 Å (Figures 2b, S22).^[^ ^9^ ^]^ The dipole moment of the optimized structure of **1au** ^+^ was 10.68 D, which was larger than that of **1au´** ^+^ (6.02 D),^[^ ^3b^ ^]^ indicating the effectively induced polarized structure for **1au** ^+^. The UV/vis absorption spectrum of **1au** ^+^‐Cl^‐^ in CH_2_Cl_2_ displayed Soret and Q bands at 391 and 507/540 nm, respectively (Figure S8a), as correlated with the TD‐DFT spectrum (Figure S25). The Soret band for **1au** ^+^‐Cl^‐^ is blue‐shifted by 6 and 10 nm compared with those of **1au´** ^+^ and tetra‐*meso*‐C_6_F_5_‐substituted porphyrin Au^III^ complex, respectively.^[^ ^3b^ ^]^ Notably, other ion pairs displayed similar absorption spectra (Figure S8a). The redox behavior of **1au** ^+^‐PF_6_^‐^ was evaluated by cyclic voltammetry (CV) in CH_2_Cl_2_ containing 0.1 M TBAPF_6_ as an electrolyte (Figure S9). The first reduction potential (versus Ag^+^/Ag) of **1au** ^+^ was estimated to be −0.66 V, which was more unlikely to be reduced by 0.11 V than that of **1au´** ^+^.^[^ ^3c^ ^]^ The fewer number of electron‐withdrawing C_6_F_5_ units induced the less electron‐deficient porphyrin core unit, as suggested by electrostatic potential mapping (ESP) (Figure S23).

**Figure 3:**
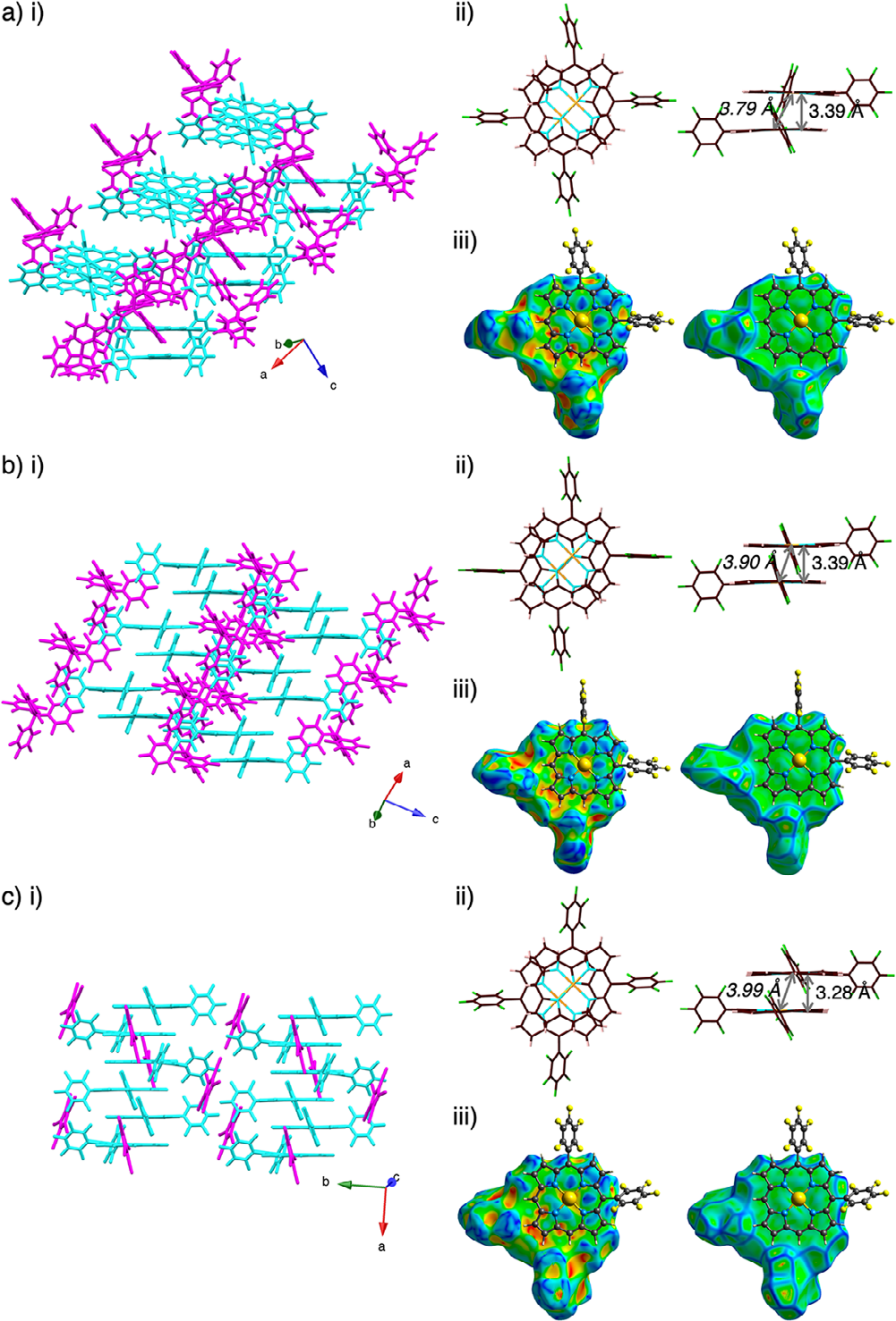
Single‐crystal X‐ray structures of a) **1au** ^+^‐FABA^–^_D_, b) **1au** ^+^‐FABA^–^_A_, and c) **1au** ^+^‐PCCp^–^ as i) packing structures, ii) top and side views of stacked dimer with stacking and Au···Au (italic) distances, and iii) Hirshfeld surface mapped with shape‐index (left) and curvedness (right) (one of the stacked dimers is shown for **1au** ^+^‐FABA^‐^_D_ and **1au** ^+^‐PCCp^‐^ as a representative). Color code in i): cyan and magenta refer to cation and anion, respectively. Atom color code in ii): brown, pink, yellow, blue, green, and orange refer to carbon, hydrogen, boron, nitrogen, fluorine, and gold, respectively.

Single crystals of **1au** ^+^‐FABA^−^ suitable for X‐ray analysis were obtained by vapor diffusion. The exact structures and solid‐state assemblies of the ion pair were elucidated by single‐crystal X‐ray analysis (Figures 3a,b, S10, S11, S14, S15),^[^ ^10^ ^]^ indicating two types of pseudopolymorphs for single crystals prepared from CH_2_Cl_2_/*n*‐hexane and CH_3_CN/water. Both the assembly modes of **1au** ^+^‐FABA^‐^_D_ (from CH_2_Cl_2_/*n*‐hexane) and **1au** ^+^‐FABA^‐^_A_ (from CH_3_CN/water) displayed the stacked dimer structures of **1au** ^+^ arranged in antiparallel fashion. The interplanar distances between dimeric cations (core 25 atoms) in **1au** ^+^‐FABA^‐^_D_ and **1au** ^+^‐FABA^‐^_A_ were 3.39/3.49 and 3.39 Å, respectively (Figures 3a,b ii). The lines passing through the Au atoms have angles of 58.9°/63.7° and 60.0°, respectively, to the mean plane of **1au** ^+^. The FABA^‐^ units were located to the side of the stacked dimer with pyrrole β‐C(−H)···F distances of 3.03–3.43 and 3.15/3.25 Å in **1au** ^+^‐FABA^‐^_D_ and **1au** ^+^‐FABA^‐^_A_, respectively, suggesting that the stacking structures were stabilized by hydrogen‐bonding interactions. Hirshfeld surface analysis^[^ ^11^ ^]^ of the stacked dimers in **1au** ^+^‐FABA^‐^_D_ and **1au** ^+^‐FABA^‐^_A_ indicated bowtie‐shaped red and blue triangles in shape‐index and flat surface in the curvedness properties, which clearly showed characteristic mapping patterns for stacking of charged π‐electronic cations (Figures 3a,b iii, S18, S19).

The arrangement of the dipolar π‐electronic cation can be modulated by counteranions. In fact, X‐ray analysis for the single crystal of **1au** ^+^‐PCCp^‐^ that was prepared by vapor diffusion of acetone/*n*‐hexane revealed a charge‐segregated assembly (Figures 3c, S12, S16). **1au** ^+^ was stacked in an antiparallel fashion similar to that of the FABA ion pair, forming a columnar structure. The interplanar distances between the stacked **1au** ^+^ units (core 25 atoms) were 3.28–3.41 Å. Pyrrole β‐H and PCCp^‐^‐N formed hydrogen bonding with C(−H)···N distances of 3.24–3.82 Å. Furthermore, the stacking structure of **1au** ^+^ was indicated by Hirshfeld surface analysis, which showed a closely stacked structure (Figure S20). Notably, identically charged π‐electronic cations can form stable stacked structures with the support of dipole–dipole interactions.

**Figure 4:**
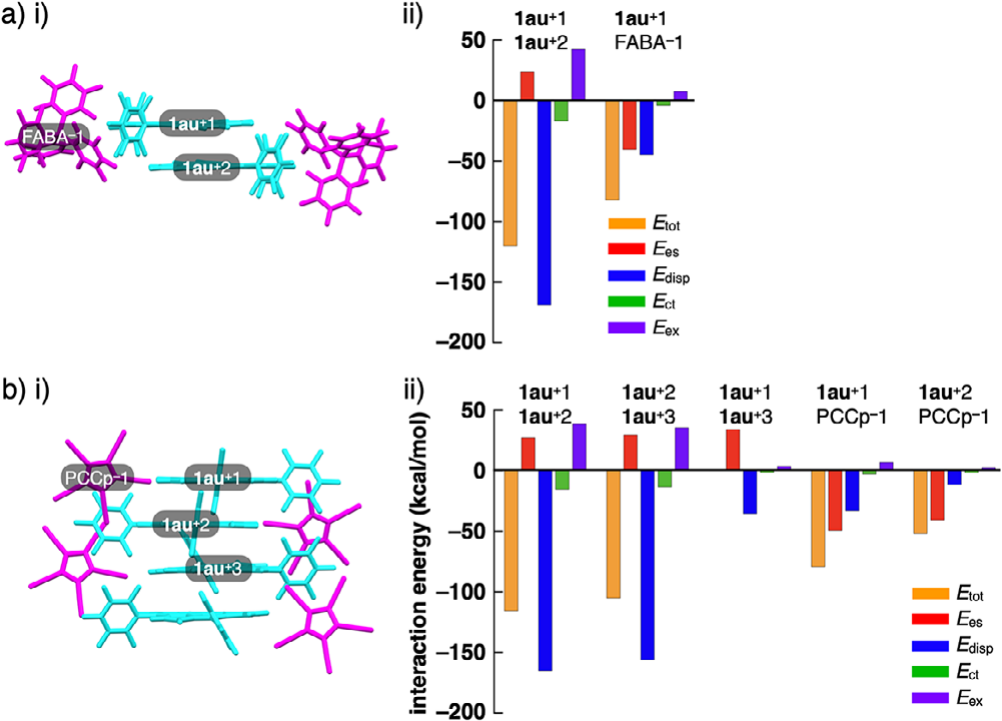
Decomposition of the total intermolecular interaction energies (*E*_tot_) of a) **1au** ^+^‐FABA^‐^_A_ and b) **1au** ^+^‐PCCp^‐^ for i) single‐crystal X‐ray structures and ii) estimated interaction energies (kcal/mol) according to EDA based on the FMO2‐MP2 method using a basis set of NOSeC‐V‐DZP with MCP with TZP for Au (see Tables S3, S4 for the complete data list). Color code in i): cyan and magenta refer to cation and anion, respectively.

The packing structures depend on intermolecular interactions, which are influenced by the electronic states and shapes of the components. Energy decomposition analysis (EDA)^[^ ^12^ ^]^ was performed to evaluate the roles of intermolecular interactions via the fragment molecular orbital (FMO) method (FMO2‐MP2) involving mixed basis sets including NOSeC‐V‐DZP with model core potential (MCP) with TZP for Au (Figures 4, S29–S32, and Tables S2–S4).^[^ ^13^ ^]^ The EDA calculations using FMO yielded *E*_es_, *E*_disp_, *E*_ex_, *E*_ct_ (energies for electrostatic, dispersion, exchange‐repulsion, and charge‐transfer forces, respectively), and *E*_tot_ (total energy).^[^ ^16^, ^17^ ^]^ In **1au** ^+^‐FABA^‐^_D_ and **1au** ^+^‐FABA^‐^_A_, the *E*_tot_ between two stacked **1au** ^+^ units (**1au** ^+^1‐**1au** ^+^2) was −119.94 and −120.08 kcal/mol, respectively (Figures 4a, S29, S30). Despite repulsive electrostatic forces (*E*_es_ = 22.45 and 23.81 kcal/mol, respectively), the dispersion forces (*E*_disp_ = ‐164.16 and ‐169.11 kcal/mol, respectively) stabilized the stacked dimer structures. The *E*_tot_ between **1au** ^+^ and FABA^‐^ (**1au** ^+^1‐FABA^‐^1) (‐93.42 and ‐81.97 kcal/mol, respectively) were smaller than those of two stacked **1au** ^+^ owing to the smaller dispersion forces (*E*_disp_ = ‐50.70 and ‐44.86 kcal/mol, respectively). Similar interaction energy balances for the stacking of **1au** ^+^ in the charge‐segregated assembly of **1au** ^+^‐PCCp^‐^ were observed (Figures 4b, S31). The *E*_tot_ between two stacked **1au** ^+^ (**1au** ^+^1‐**1au** ^+^2 and **1au** ^+^2‐**1au** ^+^3) was ‐115.63 and ‐105.19 kcal/mol derived from the electrostatic force (*E*_es_ = 26.88 and 29.47 kcal/mol, respectively) and dispersion force (*E*_disp_ = ‐165.08 and ‐156.22 kcal/mol, respectively). Notably, *E*_es_ between **1au** ^+^1 and **1au** ^+^2 was lower than that of **1au** ^+^1 and **1au** ^+^3. The reduced electrostatic repulsion between stacked **1au** ^+^ can be ascribed to the dipole–dipole interaction originating from the dipole moment of **1au** ^+^. The effective dispersion and reduced electrostatic forces stabilized the stacking of identically charged cations.

**Figure 5:**
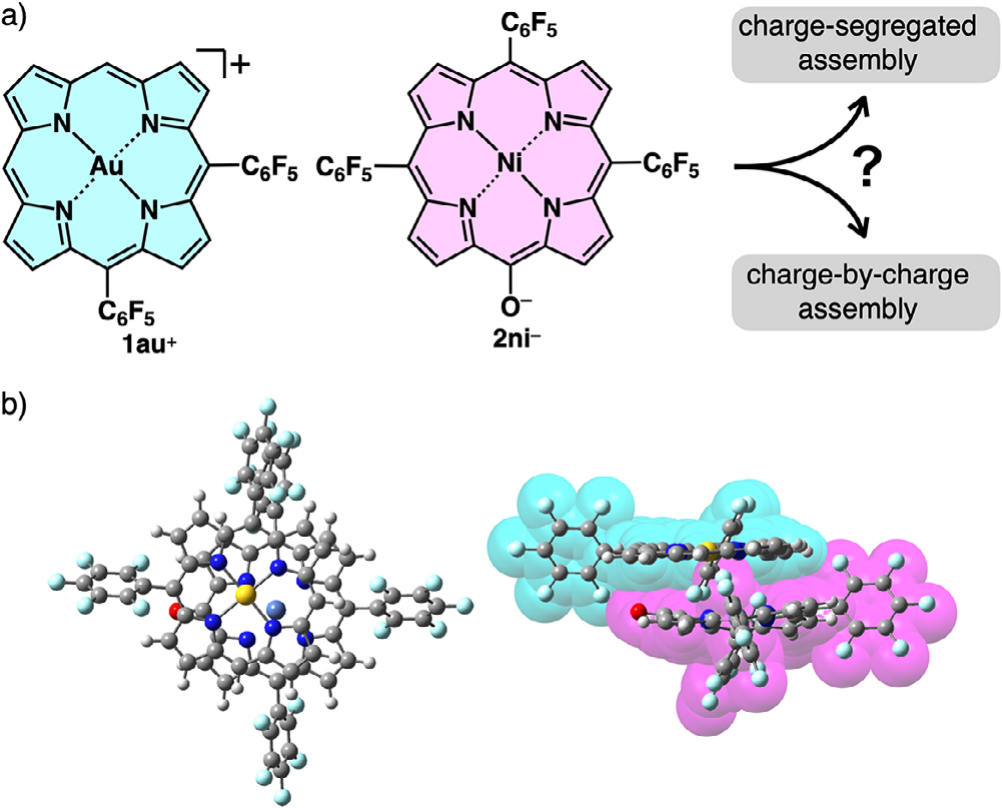
a) π‐Electronic ion pair **1au** ^+^‐2ni^‐^ and diagram for formation of ion‐pairing assemblies and b) the optimized structure of **1au** ^+^‐2ni^‐^ at GD3BJ‐B3LYP/6–31G(d,p) with LanL2DZ for Ni and Au (cyan and magenta in the side view represent cation and anion parts, respectively).

The formation of a charge‐segregated assembly through the combination with PCCp^‐^ as a π‐electronic anion further motivated the introduction of larger π‐electronic anions, such as porphyrin anions, to form ion‐pairing assemblies with modulated assembly modes (Figure 5a). Deprotonated *meso*‐hydroxyporphyrin **2ni** ^‐^ should be a suitable component in ion‐pairing assemblies, as a π‐electronic anion that is stabilized by the delocalized negative charge in the porphyrin core unit.^[^ ^3^, ^16^ ^]^ Ion‐pair metathesis of **1au** ^+^‐Cl^‐^ and Na^+^‐**2ni** ^‐^ resulted in the production of **1au** ^+^‐**2ni** ^‐^ in 92% yield (Figure S7). The ion pair was characterized by ^1^H, ^13^C, and ^19^F NMR along with ESI‐TOF‐MS. **1au** ^+^‐**2ni** ^‐^ exhibited Soret and Q bands derived from **2ni** ^‐^ at 434 and 678 nm, respectively, in addition to those of **1au** ^+^ as observed in other ion pairs (Figure S8b). Concentration‐dependent ^1^H NMR of **1au** ^+^‐**2ni** ^‐^ in CD_2_Cl_2_ exhibited the larger amount of the stacked dimer at higher concentrations (Figure S33). Upfield shifts of the *meso*‐CH and proximal β‐H signals of **1au** ^+^ and the β‐H signals of **2ni** ^‐^ close to the C−O^‐^ unit are derived from the shielded effects by the formation of π‐stacked ion pair (*π‐sip*), as also suggested by the optimized structure (Figures 5b, S28). The curve fitting for the chemical shifts provided a hetero‐dimerization constant (*K*_dim_) of 320 M^−1^ for stacking **1au** ^+^ and **2ni** ^‐^. The less effective formation of the *π‐sip* than **1au´** ^+^‐**2ni** ^‐^ (2.8 × 10^5^ M^−1^)^[^ ^3c^ ^]^ can be attributed to the larger dipole of **1au** ^+^ in a nearly parallel arrangement with that of **2ni** ^‐^ and the less electron‐withdrawing moieties in **1au** ^+^. The smaller *K*_dim_ value suggested that **1au** ^+^ would form charge‐segregated structures in combination with suitable π‐electronic anions.

**Figure 6:**
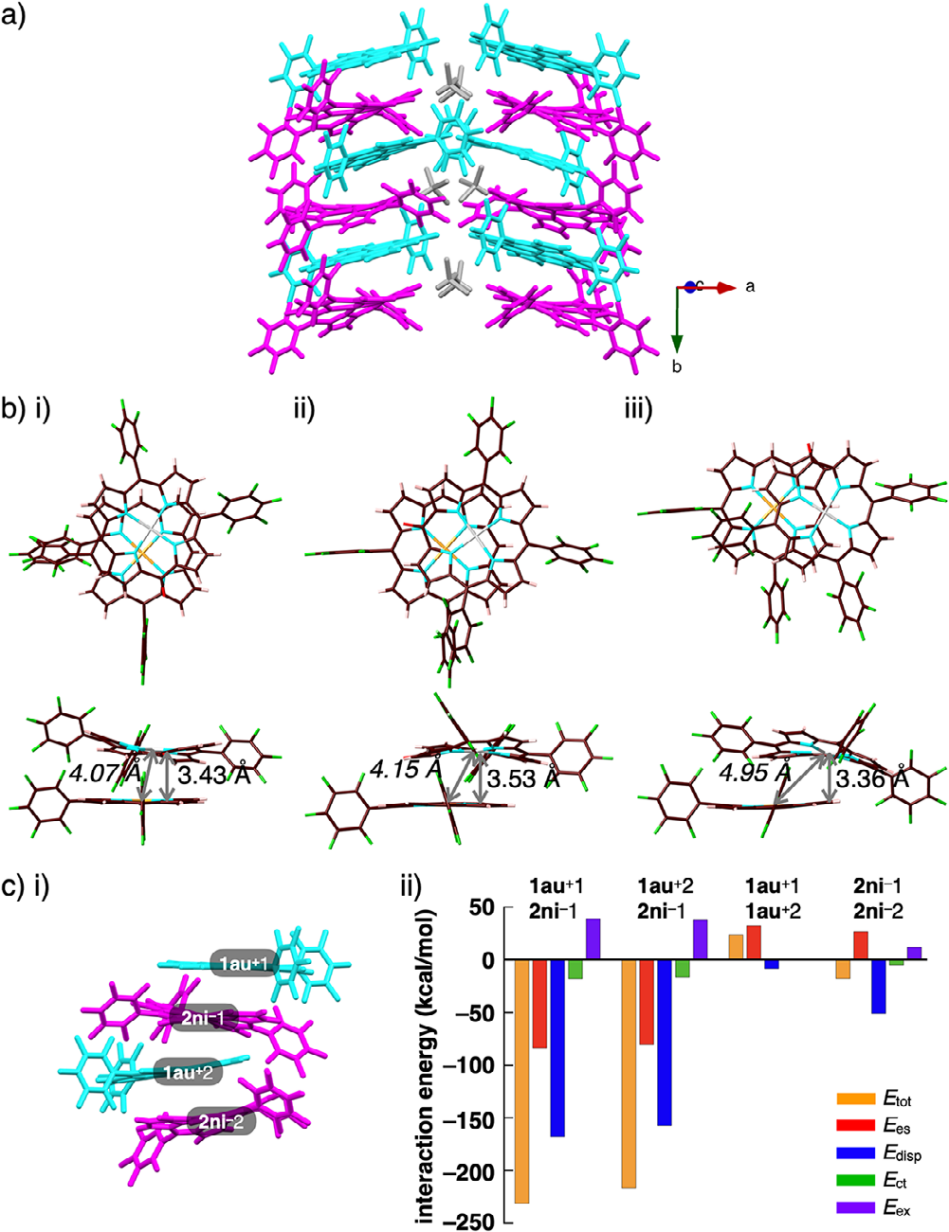
Single‐crystal X‐ray structure of **1au** ^+^‐**2ni** ^‐^ as a) packing structure and b) top and side views of three stacking modes with stacking and Au···Au (italic) distances, and c) decomposition of the total intermolecular interaction energies (*E*_tot_) of **1au** ^+^‐**2ni** ^‐^ for i) single‐crystal X‐ray structure and ii) estimated interaction energies (kcal/mol) according to EDA based on the FMO2‐MP2 method using a basis set of NOSeC‐V‐DZP with MCP with TZP for Ni and Au (see Table S5 for the complete data list). Color code in a) and c) i): cyan, magenta, and gray refer to cation, anion, and CH_3_CN, respectively. Atom color code in b): brown, pink, blue, red, green, gray, and orange refer to carbon, hydrogen, nitrogen, oxygen, fluorine, nickel, and gold, respectively.

Ion‐pairing assembly of **1au** ^+^‐**2ni** ^‐^ was revealed by X‐ray analysis for a single crystal prepared by vapor diffusion of CH_3_CN‐THF/*n*‐hexane. In contrast to the charge‐segregated assembly of the PCCp^‐^ ion pair, **1au** ^+^‐**2ni** ^‐^ exhibited a charge‐by‐charge assembly, with stacking distances of 3.36–3.53 Å between the mean planes (core 25 atoms) of **1au** ^+^ and **2ni** ^‐^ (Figures 6a, S13, S17). **1au** ^+^ and **2ni** ^‐^, with a dipole moment of 7.52 D in the optimized structure, were arranged in three different stacking modes via the complex contributions from fundamental intermolecular forces (Figure 6b). CH_3_CN molecules formed hydrogen bonding with the *meso*‐C−O^‐^ of **2ni** ^‐^ (Figure 6a). Hirshfeld surface analysis of **1au** ^+^‐**2ni** ^‐^ showed characteristic shape‐index and curvedness mapping patterns for stacking of π‐electronic systems, revealing the formation of the *π‐sip* (Figure S21). In contrast to the electrostatic repulsion in the stacking structures of **1au** ^+^ in **1au** ^+^‐FABA^‐^ and **1au** ^+^‐PCCp^‐^, an attractive electrostatic force was observed in the charge‐by‐charge assembly of **1au** ^+^‐**2ni** ^‐^ (Figures 6c, S32, and Table S5). The EDA calculation for the crystal structure of **1au** ^+^‐**2ni** ^‐^ indicated *E*_tot_ of −231.15 and −216.99 kcal/mol for **1au** ^+^1‐**2ni** ^‐^1 and **1au** ^+^2‐**2ni** ^‐^1, respectively, derived mainly from an attractive *E*_es_ of ‐83.67 and ‐80.59 kcal/mol and *E*_disp_ of ‐168.13 and ‐157.61 kcal/mol, respectively, suggesting stabilization of the charge‐by‐charge assembly through ^*i*^π–^*i*^π interactions. Steric *meso*‐C_6_F_5_ moieties in **1au** ^+^ and **2ni** ^‐^ may induce less effective dispersion forces between identically charged π‐electronic systems, resulting in the formation of the charge‐by‐charge assembly.

## 3 Conclusion

A porphyrin Au^III^ complex comprising electron‐withdrawing units at the 5,10‐positions was synthesized as a polarized cation, and counteranions were exchanged through ion‐pair metathesis. Ion‐pairing assemblies in the single crystals, revealed by X‐ray analysis, suggested the stacking of the dipolar cations via attractive dipole–dipole interactions. In particular, the ion pair with PCCp^‐^ formed a charge‐segregated assembly based on a columnar structure of the dipolar cations stacked in antiparallel fashion. By contrast, the ion pair with a larger π‐electronic porphyrin anion formed a charge‐by‐charge assembly, driven by attractive electrostatic and dispersion forces (^*i*^π–^*i*^π interactions). Although the formation of charge‐segregated assemblies using larger π‐electronic anions by controlling the interionic interactions is challenging, the use of dipole–dipole interactions between identically charged π‐electronic systems is an effective strategy for reducing the electrostatic repulsion. Further investigations of the combinations of ion pairs comprising dipolar π‐electronic cations would lead to the formation of charge‐segregated assemblies for materials with enhanced electric conductivity properties.^[^ ^17^ ^]^

## 4 Experimental Section

### Crystallographic data

Deposition numbers 2430240–2430243 contain the supplementary crystallographic data for this paper. These data are provided free of charge by the joint Cambridge Crystallographic Data Centre and Fachinformationszentrum Karlsruhe Access Structures service.

## Conflict of Interest

The authors declare no conflict of interest.

## Supporting information



Supporting Information

## Data Availability

The data that support the findings of this study are available in the supplementary material of this article.
