# Supplementary material for: Ion‐Pairing Assemblies of Polarized Charged Porphyrin
Source: Chemistry. 2025 Aug 7;31(49):e02225. doi: 10.1002/chem.202502225 (PMC12405815; doi:10.1002/chem.202502225)
Supplement: Supplementary file 1 — Supporting Information [file CHEM-31-e02225-s001.pdf]

Supporting Information

Ion-Pairing Assemblies of Polarized Charged Porphyrin

Ryo Kitayama, Yohei Haketa, Yuto Maruyama, Masaki Fujita, Hiroki Tanaka, Hiroki Horita, and Hiromitsu Maeda*

Department of Applied Chemistry, College of Life Sciences, Ritsumeikan University, Kusatsu 525–8577, Japan, Fax: +81 77 561 3729; Tel: +81 77 561 5969; E-mail: maedahir@ph.ritsumei.ac.jp

Table of Contents

1. Synthetic procedures and spectroscopic data S2
Figure S1–7 ^{1}H, ^{13}C, and ^{19}F NMR spectra. S5
Figure S8 UV/vis absorption spectra. S18
Figure S9 Cyclic voltammogram. S18
2. X-ray crystallographic data S19
Figure S10–13 Ortep drawings. S20
Figure S14–17 Packing structures. S23
Figure S18–21 Hirshfeld analysis. S26
3. Theoretical studies S31
Figure S22 Optimized structure. S31
Figure S23 Electrostatic potential (ESP) mapping. S31
Figure S24 Molecular orbitals (HOMO/LUMO). S32
Figure S25 Theoretical UV/vis absorption spectrum. S33
Figure S26 NICS values. S33
Figure S27 ACID plots. S33
Figure S28 Evaluation of dipole–dipole interactions in π-stacked ion pairs. S34
Figure S29–32 Single-crystal X-ray structures for EDA calculations. S34
Cartesian coordination of optimized structures S37
4. Solution-state properties S40
Figure S33 Concentration-dependent ^{1}H NMR spectra. S40

1. Synthetic procedures and spectroscopic data

General procedures. Starting materials were purchased from FUJIFILM Wako Pure Chemical Corp., Nacalai Tesque Inc., Tokyo Chemical Industry Co., Ltd., and Sigma-Aldrich Co. and were used without further purification unless otherwise stated. Heating reaction was conducted using oil bath. NMR spectra used in the characterization of products were recorded on a JEOL ECA-600 600 MHz spectrometer. All NMR spectra were referenced to solvent. Matrix-assisted laser desorption ionization time-of-flight mass spectrometry (MALDI-TOF-MS) was recorded on a Shimadzu Axima-CFRplus. High-resolution (HR) electrospray ionization mass spectrometry (ESI-MS) was recorded on a BRUKER microTOF using ESI-TOF method. TLC analyses were carried out on aluminum sheets coated with silica gel 60 (Merck 5554). Column chromatography was performed on Wakogel C-300 and Merck silica gel 60 and 60H.

Au^{III} complex of 5,10-bis(pentafluorophenyl)-porphyrin as a OTf^- ion pair, $1au^+$-OTf^-. To 5,10-bis(pentafluorophenyl)porphyrin[S1] **1** (30.3 mg, 47.1 μmol) and NaOAc (63.0 mg, 0.768 mmol) in $C_2H_4Cl_2$ (60 mL) heated at 60 °C were added $HAuCl_4{\cdot}4H_2O$ (78.9 mg, 191 μmol) and AgOTf (196.9 mg, 0.767 mmol) in THF (20 mL). The reaction mixture was heated to 80 °C and was stirred under N_2 for 2 h. After the solution was evaporated, the residue was chromatographed over flash silica gel column (10% $MeOH/CH_2Cl_2$) and was recrystallized from CH_2Cl_2/*n*-hexane to afford **$1au^+$**-OTf^- (22.7 mg, 23.0 μmol, 49%) as a red solid. R_f = 0.30 (10% $MeOH/CH_2Cl_2$). ^1H NMR (600 MHz, DMSO-d_6, 20 °C): δ(ppm) 11.40 (s, 2H, β-CH), 10.25 (d, J = 4.8 Hz, 2H, β-CH), 10.07 (s, 2H, β-CH), 10.00 (s, 2H, *meso*-CH), 9.93 (d, J = 4.8 Hz, 2H, β-CH). $^{13}C\{^1H\}$ NMR (151 MHz, DMSO-d_6, 20 °C): δ(ppm) 146.41 (dm, $J_{13C\text{–}19F}$ = 247 Hz), 142.60 (dm, $J_{13C\text{–}19F}$ = 255 Hz), 137.92 (dm, $J_{13C\text{–}19F}$ = 253 Hz), 136.35, 136.24, 135.98, 135.92, 135.02, 133.84, 132.65, 131.78, 120.70 (dm, $J_{13C\text{–}19F}$ = 322 Hz), 112.59, 109.87, 104.58. ^{19}F NMR (564 MHz, DMSO-d_6, 20 °C): δ(ppm) –67.45 (s, 3F), –128.59 (d, J = 19.7 Hz, 4F), –142.47 (t, J = 19.7 Hz, 2F), –151.53 (t, J = 23.1 Hz, 4F). UV/vis (CH_2Cl_2, λ_{max}[nm] (ε, 10^5 $M^{-1}cm^{-1}$)): 391 (1.6), 508 (0.6), 542 (0.6). HRMS (ESI-TOF) *m/z*: [M – CF_3O_3S]$^+$ Calcd for $C_{32}H_{10}AuF_{10}N_4^+$ 837.0406; found 837.0406. [M – $C_{32}H_{10}AuF_{10}N_4$]$^-$ Calcd for $CF_3O_3S^-$ 148.9526; found 148.9526.

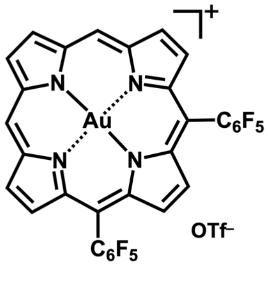

Au^{III} complex of 5,10-bis(pentafluorophenyl)-porphyrin as a Cl^- ion pair, $1au^+$-Cl^-. A solution of **$1au^+$**-OTf^- was chromatographed over ion-exchanged resin (Amberlite IRA402BL Cl, MeOH) and silica gel column (Wakogel C-300, 10% $MeOH/CH_2Cl_2$) and was recrystallized from acetone/*n*-hexane to afford **$1au^+$**-Cl^- (11.1 mg, 12.7 μmol, 63%) as a red solid. R_f = 0.11 (10% $MeOH/CH_2Cl_2$). ^1H NMR (600 MHz, DMSO-d_6, 20 °C): δ(ppm) 11.52 (s, 2H, *meso*-CH), 10.27 (d, J = 3.6 Hz, 2H, β-CH), 10.17 (s, 2H, β-CH), 9.98 (s, 2H, β-CH), 9.94 (d, J = 4.8 Hz, 2H, β-CH). $^{13}C\{^1H\}$ NMR (151 MHz, DMSO-d_6, 20 °C): δ(ppm) 146.41 (dm, $J_{13C\text{–}19F}$ = 237 Hz), 142.68 (dm, $J_{13C\text{–}19F}$ = 225 Hz), 137.94 (dm, $J_{13C\text{–}19F}$ = 249 Hz), 136.49, 136.05, 135.96, 135.06, 134.05, 132.64, 131.81, 112.60, 110.04, 104.60. ^{19}F NMR (564 MHz, DMSO-d_6, 20 °C): δ(ppm) –128.66 (d, J = 25.9 Hz, 4F), –142.33 (t, J = 22.8 Hz, 2F), –151.49 (t, J = 23.1 Hz, 4F). UV/vis (CH_2Cl_2, λ_{max}[nm] (ε, 10^5 $M^{-1}cm^{-1}$)): 391 (3.3), 507 (0.17), 542 (0.12). MALDI-TOF-MS: *m/z* (%): (pos) 837.0 (100). Calcd for $C_{32}H_{10}AuF_{10}N_4$ ([M – Cl]$^+$): 837.51. (neg) 34.95 (100). Calcd for Cl ([M – $C_{32}H_{10}AuF_{10}N_4$]$^-$): 35.11. HRMS (ESI-TOF) *m/z*: [M – Cl]$^+$ Calcd for $C_{32}H_{10}AuF_{10}N_4^+$ 837.0406; found 837.0406. HRMS (ESI-TOF) for Cl^- could not be measured due to the measurement limitation for low molecular weight.

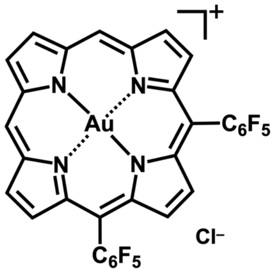

Au^{III} complex of 5,10-bis(pentafluorophenyl)-porphyrin as a BF_4^- ion pair, $1au^+$-BF_4^-. To **$1au^+$**-Cl^- (10.0 mg, 11.4 μmol) in CH_3CN/MeOH (3:1, 20 mL) was added $AgBF_4$ (6.6 mg, 34.2 μmol) in MeOH (5 mL). The reaction mixture was stirred at r.t. under N_2 for 30 min. After the solution was evaporated, the residue was chromatographed over flash silica gel column (10% $MeOH/CH_2Cl_2$) and was recrystallized from CH_2Cl_2/*n*-hexane to afford **$1au^+$**-BF_4^- (8.6 mg, 5.7 μmol, 50%) as a red solid. R_f = 0.30 (10% $MeOH/CH_2Cl_2$). ^1H NMR (600 MHz, DMSO-d_6, 20 °C): δ(ppm) 11.35 (s, 2H, *meso*-CH), 10.22 (d, J = 4.8 Hz, 2H, β-CH), 10.01 (s, 2H, β-CH), 10.00 (s, 2H, β-CH). $^{13}C\{^1H\}$ NMR (151 MHz, DMSO-d_6, 20 °C): δ(ppm) 146.44 (dm, $J_{13C\text{–}19F}$ = 244 Hz), 142.66 (dm, $J_{13C\text{–}19F}$ = 241 Hz), 137.97 (dm, $J_{13C\text{–}19F}$ = 252 Hz), 136.34, 136.18, 136.00, 135.96, 135.03, 133.76, 132.68, 131.80, 112.60, 109.80, 104.65. ^{19}F NMR (564 MHz, DMSO-d_6, 20 °C): δ(ppm) –128.57 (d, J = 19.7 Hz, 4F), –137.96 (d, J = 32.7 Hz, 4F), –142.26 (t, J = 23.1 Hz, 2F), –151.45 (t, J = 22.8 Hz, 4F). UV/vis (CH_2Cl_2, λ_{max}[nm] (ε, 10^5 $M^{-1}cm^{-1}$)): 391 (2.0), 506 (0.1), 541 (0.1). HRMS (ESI-TOF) *m/z*: [M – BF_4]$^+$ Calcd for

$C_{32}H_{10}AuF_{10}N_4^+$ 837.0406; found 837.0406; [M – $C_{32}H_{10}AuF_{10}N_4]^-$ Calcd for BF_4^- 87.0035; found 87.0035.

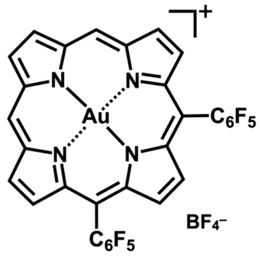

Au^{III} complex of 5,10-bis(pentafluorophenyl)-porphyrin as a PF_6^- ion pair, 1au$^+$-PF_6^-. To **1au**$^+$-Cl$^-$ (10.0 mg, 11.4 μmol) in CH_3CN/MeOH (3:1, 20 mL) was added $AgPF_6$ (8.6 mg, 34.2 μmol) in MeOH (5 mL). The reaction mixture was stirred at r.t. under N_2 for 30 min. After the solution was evaporated, the residue was chromatographed over flash silica gel column (10% MeOH/CH_2Cl_2) and was recrystallized from CH_2Cl_2/*n*-hexane to afford **1au**$^+$-PF_6^- (8.6 mg, 8.1 μmol, 71%) as a red solid. R_f = 0.31 (10% MeOH/CH_2Cl_2). ^{1}H NMR (600 MHz, DMSO-d_6, 20 °C): δ(ppm) 11.44 (s, 2H, *meso*-CH), 10.26 (d, J = 4.8 Hz, 2H, β-CH), 10.11 (s, 2H, β-CH), 10.00 (s, 2H, β-CH), 9.95 (d, J = 4.8 Hz, 2H, β-CH). ^{13}C{^{1}H} NMR (151 MHz, DMSO-d_6, 20 °C): δ (ppm) 146.38 (dm, $J_{13C-19F}$ = 243 Hz), 142.65 (dm, $J_{13C-19F}$ = 244 Hz), 137.90 (dm, $J_{13C-19F}$ = 244 Hz), 136.37, 136.28, 135.98, 135.91, 135.00, 133.87, 132.62, 131.75, 112.56, 109.87, 104.55. ^{19}F NMR (564 MHz, DMSO-d_6, 20 °C): δ(ppm) –59.85 (d, J = 709.5 Hz, 6F), –128.59 (d, J = 19.7 Hz, 4F), –142.47 (t, J = 23.1 Hz, 2F), –151.53 (t, J = 23.1 Hz, 2F). UV/vis (CH_2Cl_2, λ_{max}[nm] (ε, 10^5 $M^{-1}cm^{-1}$)): 391 (2.3), 507 (0.10), 542 (0.10). HRMS (ESI-TOF) *m/z*: [M – $PF_6]^+$ Calcd for $C_{32}H_{10}AuF_{10}N_4^+$ 837.0406; found 837.0406. [M – $C_{32}H_{10}AuF_{10}N_4]^-$ Calcd for PF_6^- 144.9647; found 144.9647.

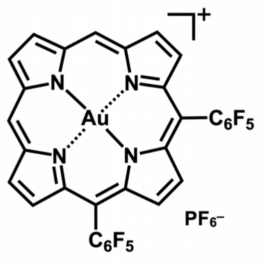

Au^{III} complex of 5,10-bis(pentafluorophenyl)-porphyrin as a FABA$^-$ ion pair, 1au$^+$-FABA$^-$. To **1au**$^+$-Cl$^-$ (10.0 mg, 11.4 μmol) in CH_3CN/MeOH (3:1, 20 mL) was added $LiB(C_6F_5)_4$ (LiFABA) (39.1 mg, 57.0 μmol) in MeOH (5 mL). The reaction mixture was stirred at r.t. under N_2 for 30 min. After the solution was evaporated, the residue was chromatographed over flash silica gel column (5% MeOH/CH_2Cl_2) and was recrystallized from CH_2Cl_2/*n*-hexane to afford **1au**$^+$-FABA$^-$ (8.6 mg, 5.7 μmol, 50%) as a red solid. R_f = 0.50 (5% MeOH/CH_2Cl_2). ^{1}H NMR (600 MHz, CD_3CN, 20 °C): δ(ppm) 11.31 (s, 2H, *meso*-CH), 10.10 (d, J = 4.8 Hz, 2H, β-CH), 10.04 (s, 2H, β-CH), 9.70 (s, 2H, β-CH), 9.69 (d, J = 4.8 Hz, 2H, β-CH). ^{13}C{^{1}H} NMR (151 MHz, CD_3CN, 20 °C): δ(ppm) 149.08 (dm, $J_{13C-19F}$ = 248 Hz), 147.87 (dm, $J_{13C-19F}$ = 255 Hz), 144.51 (dm, $J_{13C-19F}$ = 255 Hz), 139.56 (dm, $J_{13C-19F}$ = 244 Hz), 139.27 (dm, $J_{13C-19F}$ = 250 Hz), 137.27 (dm, $J_{13C-19F}$ = 255 Hz), 137.15, 136.88, 136.43, 135.57, 135.35, 133.39, 132.87, 132.37, 113.58 (t, $J_{13C-19F}$ = 18.3 Hz), 109.12, 106.76 (a signal is missing due to overlapping with another signal). ^{19}F NMR (564 MHz, CD_3CN, 20 °C): δ(ppm) –136.98 (s, 8F), –142.62 (d, J = 12.9 Hz, 4F), –156.65 (t, J = 19.7 Hz, 2F), –166.13 (t, J = 19.5 Hz, 4F), –167.09 (t, J = 19.5 Hz, 4F), –171.52 (d, J = 19.2 Hz, 8F). UV/vis (CH_2Cl_2, λ_{max}[nm] (ε, 10^5 $M^{-1}cm^{-1}$)): 391 (2.3), 507 (0.10), 542 (0.09). HRMS (ESI-TOF) *m/z*: [M –$C_{24}BF_{20}]^+$ Calcd for $C_{32}H_{10}AuF_{10}N_4^+$ 837.0406; found 837.0406. [M – $C_{32}H_{10}AuF_{10}N_4]^-$ Calcd for $C_{24}BF_{20}^-$ 678.9779; found 678.9779. This compound was further characterized by single-crystal X-ray analysis.

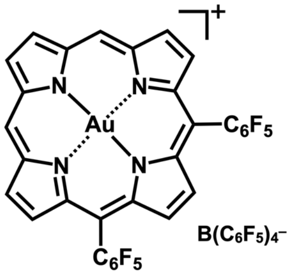

Au^{III} complex of 5,10-bis(pentafluorophenyl)-porphyrin as a PCCp$^-$ ion pair, 1au$^+$-PCCp$^-$. To **1au**$^+$-Cl$^-$ (5.0 mg, 5.7 μmol) in CH_3CN/MeOH (3:1, 20 mL) was added sodium pentacyanocyclopentadienide (NaPCCp)[S2] (3.8 mg, 18.0 μmol) in MeOH (5 mL). The reaction mixture was stirred at r.t. under N_2 for 30 min. After the solution was evaporated, the residue was chromatographed over flash silica gel column (10% MeOH/CH_2Cl_2) and was recrystallized from CH_2Cl_2/*n*-hexane to afford **1au**$^+$-PCCp$^-$ (3.8 mg, 3.7 μmol, 64%) as a red solid. R_f = 0.50 (10% MeOH/CH_2Cl_2). ^{1}H NMR (600 MHz, DMSO-d_6, 20 °C): δ(ppm) 11.49 (s, 2H, *meso*-CH), 10.27 (d, J = 5.4 Hz, 2H, β-CH), 10.16 (s, 2H, β-CH), 9.99 (s, 2H, β-CH), 9.95 (d, J = 5.4 Hz, 2H, β-CH). ^{13}C{^{1}H} NMR (151 MHz, DMSO-d_6, 20 °C): δ (ppm) 146.34 (dm, $J_{13C-19F}$ = 247 Hz), 142.52 (dm, $J_{13C-19F}$ = 243 Hz), 137.86 (dm, $J_{13C-19F}$ = 260 Hz), 136.41, 136.36, 136.00, 135.92, 135.00, 133.95, 132.61, 131.77, 112.89, 112.57, 109.92, 104.55, 101.59. ^{19}F NMR (564 MHz, DMSO-d_6, 20 °C): δ(ppm) –128.54 (d, J = 19.7 Hz, 4F), –142.48 (t, J = 22.8 Hz, 2F), –151.55 (t, J = 19.7 Hz, 4F). UV/vis (CH_2Cl_2, λ_{max}[nm] (ε, 10^5 $M^{-1}cm^{-1}$)): 391 (2.5), 508 (0.08), 541 (0.07). HRMS (ESI-TOF) *m/z*: [M – $C_{10}N_5]^+$ Calcd for $C_{32}H_{10}AuF_{10}N_4^+$ 837.0406; found 837.0406. [M – $C_{32}H_{10}AuF_{10}N_4]^-$ Calcd for $C_{10}N_5^-$ 190.0159; found 190.0159. This compound was further characterized by single-crystal X-ray analysis.

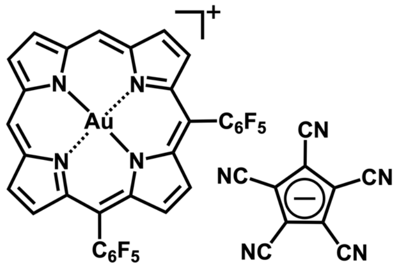

Au^{III} complex of 5,10-bis(pentafluorophenyl)porphyrin as a 2ni^{-} ion pair, 1au^{+}-2ni^{-}. To a CH_2Cl_2 (10 mL) of 5-hydroxy-10,15,20-tris(pentafluorophenyl)-3-phenylporphyrin Ni^{II} complex[S3] **2ni** (8.8 mg, 10.0 μmol) was treated with 10 mL of 10% NaOH aqueous solution.[S4] Then, **1au**$^{+}$-Cl^{-} (8.8 mg, 10.0 μmol) in CH_2Cl_2 (20 mL) was added to the biphasic solution and was washed with water to remove NaCl. The reaction mixture was evaporated to dryness. The crude product was recrystallized from CH_2Cl_2/*n*-hexane to afford **1au**$^{+}$-**2ni**$^{-}$ (15.8 mg, 9.2 μmol, 92%) as a brawn solid. ^{1}H NMR (600 MHz, CD_3CN, 20 °C): δ (ppm) 9.53 (s, 2H, *meso*-CH (**1au**$^{+}$)), 9.09 (d, J = 4.8 Hz, 2H, β-CH (**1au**$^{+}$)), 8.50 (d, J = 4.8 Hz, 2H, β-CH (**1au**$^{+}$)), 7.85 (m, 4H, **1au**$^{+}$), 7.70 (s, 2H, β-CH (**2ni**$^{-}$)), 6.37 (d, J = 3.6 Hz, 2H, β-CH (**2ni**$^{-}$)), 6.07 (s, 2H, β-CH (**2ni**$^{-}$)), 5.63 (s, 2H, β-CH (**2ni**$^{-}$)). ^{13}C{^{1}H} NMR (151 MHz, CD_3CN, 20 °C): δ (ppm) 162.60, 148.56, 148.28, 147.32, 147.18, 147.12, 146.92, 146.69, 145.59, 145.56, 145.19, 143.45, 143.22, 141.72, 140.29, 139.33, 138.68, 138.59, 137.70, 137.61, 136.12, 135.59, 135.00, 134.31, 134.13, 132.44, 132.40, 132.06, 130.92, 128.80, 127.95, 127.65, 121.26, 115.33, 113.66, 106.09, 105.87, 105.84, 103.86, 92.75 (several signals are missing, and the coupling with fluorine could not be assigned due to overlapping with other signals). ^{19}F NMR (564 MHz, CD_3CN, 20 °C): δ (ppm) –137.99 (d, J = 19.7 Hz, 4F), –140.27 (d, J = 19.7 Hz, 2F), –140.90 (d, J = 19.7 Hz, 4F), –153.30 (t, J = 19.7 Hz, 2F,), –157.25 (t, J = 19.7 Hz, 3F), –162.85 (d, J = 19.7 Hz, 4F), –164.54 (s, 2F), –164.95 (t, J = 13.0 Hz, 4F). UV/vis (CH_2Cl_2, λ_{max}[nm] (ε, 10^5 $M^{-1}cm^{-1}$)): 391 (1.63), 434 (0.48), 509 (0.07), 541 (0.07), 678 (0.10). HRMS (ESI-TOF) *m/z*: [M – $C_{38}H_8F_{15}N_4NiO$]$^{+}$ Calcd for $C_{32}H_{10}AuF_{10}N_4^{+}$ 837.0406; found 837.0406. [M – $C_{32}H_{10}AuF_{10}N_4$]$^{-}$ Calcd for $C_{38}H_8F_{15}N_4NiO^{-}$ 978.9818; found 978.9818. This compound was further characterized by single-crystal X-ray analysis.

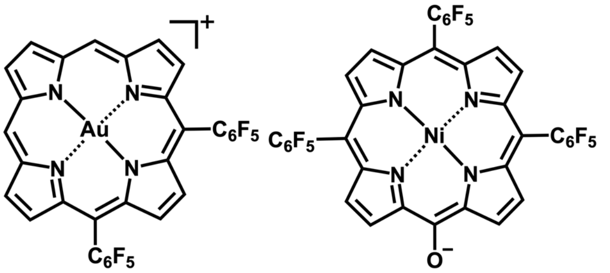

[S1] A. Osuka, T. Tanaka, K. Ueta, S. Ikeda, N. Aratani, *J. Porphyrin Phthalocyanines* **2017**, *21*, 803–810.

[S2] a) O. W. Webster, *J. Am. Chem. Soc.* **1965**, *87*, 1820–1821; b) T. Sakai, S. Seo, J. Matsuoka, Y. Mori, *J. Org. Chem.* **2013**, *78*, 10978–10985.

[S3] a) C. Stähler, D. Shimizu, K. Yoshida, K. Furukawa, R. Herges, A. Osuka, *Chem. Eur. J.* **2017**, *23*, 7217–7220; b) Y. Sasano, N. Yasuda, H. Maeda, *Dalton Trans.* **2017**, *46*, 8924–8928.

[S4] a) Y. Sasano, H. Tanaka, Y. Haketa, Y. Kobayashi, Y. Ishibashi, T. Morimoto, R. Sato, Y. Shigeta, N. Yasuda, T. Asahi, H. Maeda, *Chem. Sci.* **2021**, *12*, 9645–9657; b) H. Tanaka, Y. Kobayashi, K. Furukawa, Y. Okayasu, S. Akine, N. Yasuda, H. Maeda, *J. Am. Chem. Soc.* **2022**, *144*, 21710–21718.

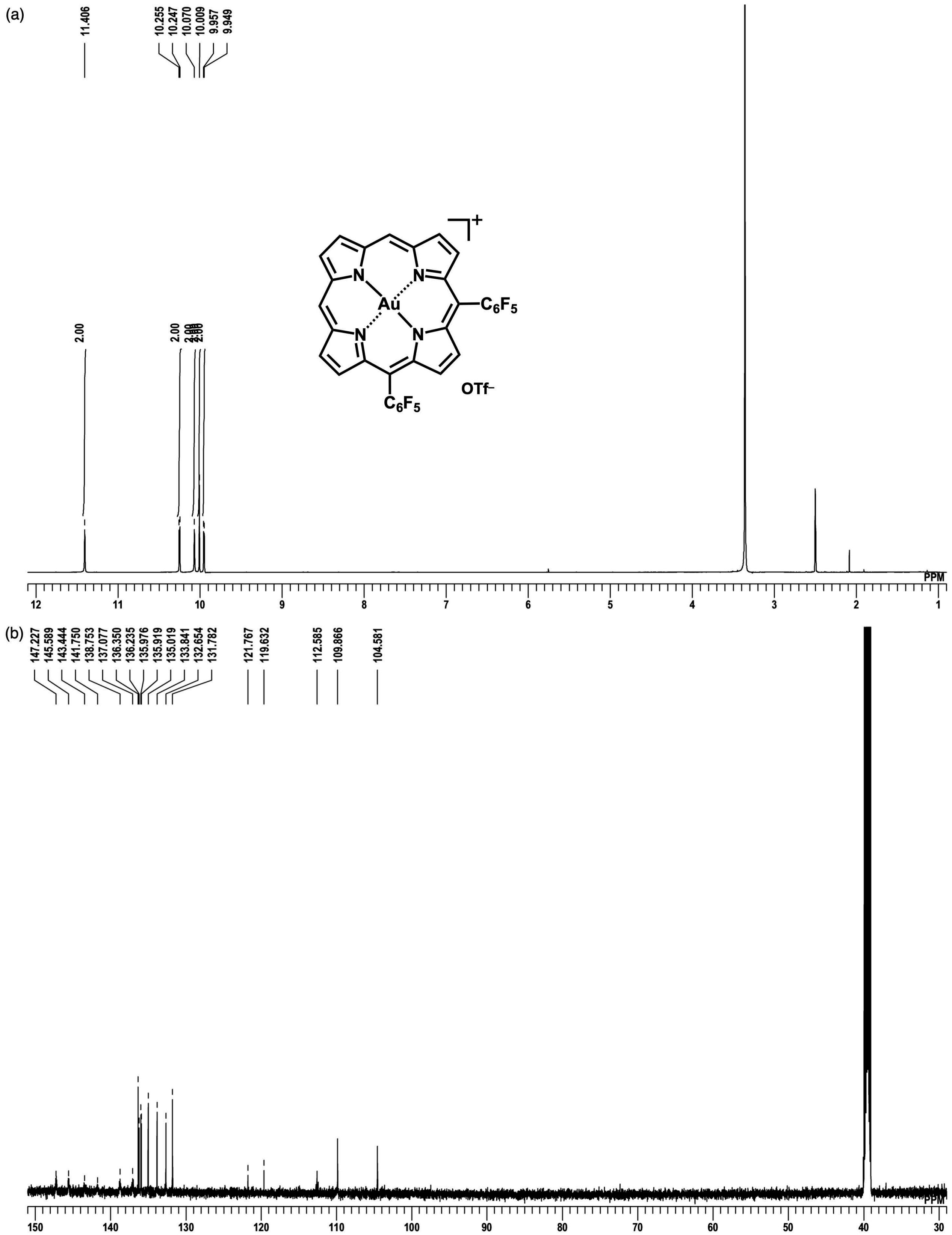

Figure S1 (a) ^{1}H NMR (600 MHz), (b) $^{13}C\{^{1}H\}$ NMR (151 MHz), and (c) ^{19}F NMR (564 MHz) spectra of **1au**$^{+}$-OTf^{-} in CD_3CN at 20 °C.

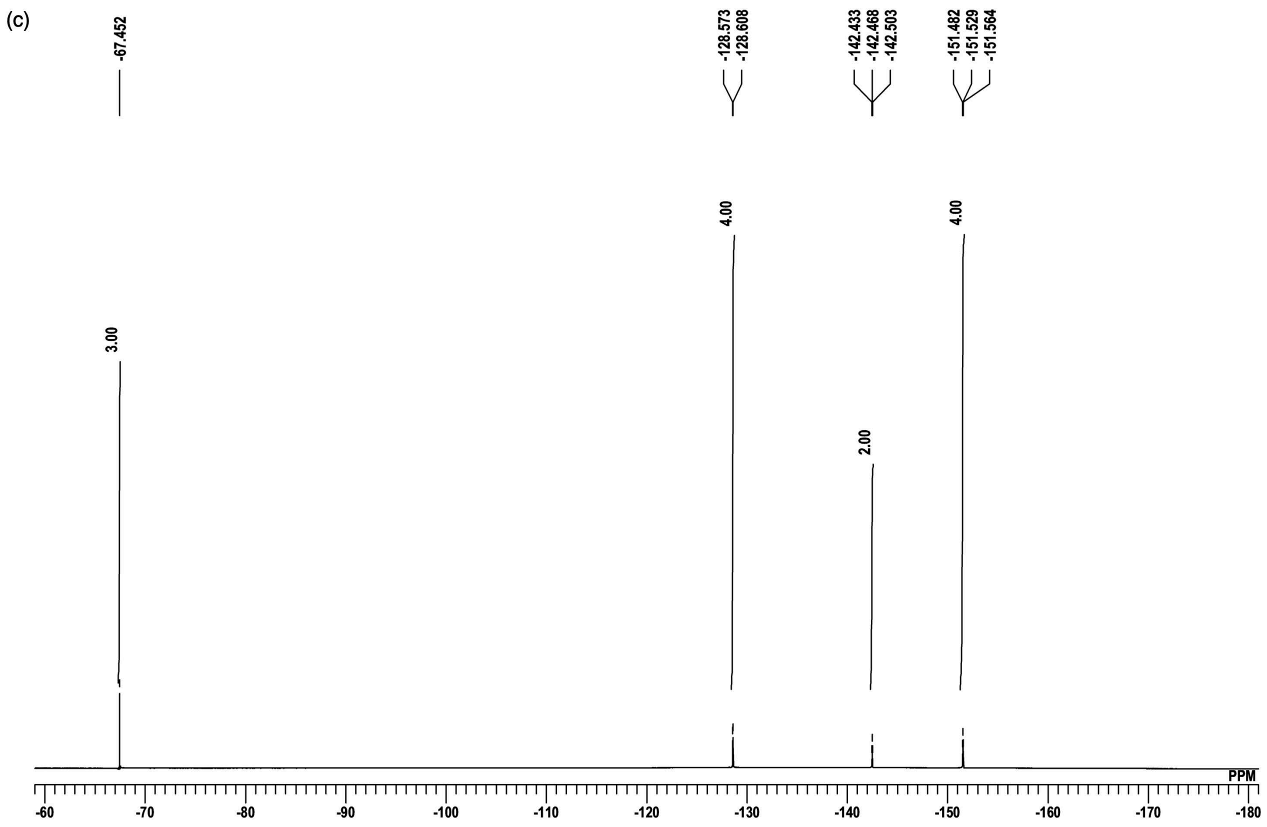

Figure S1 (Continued)

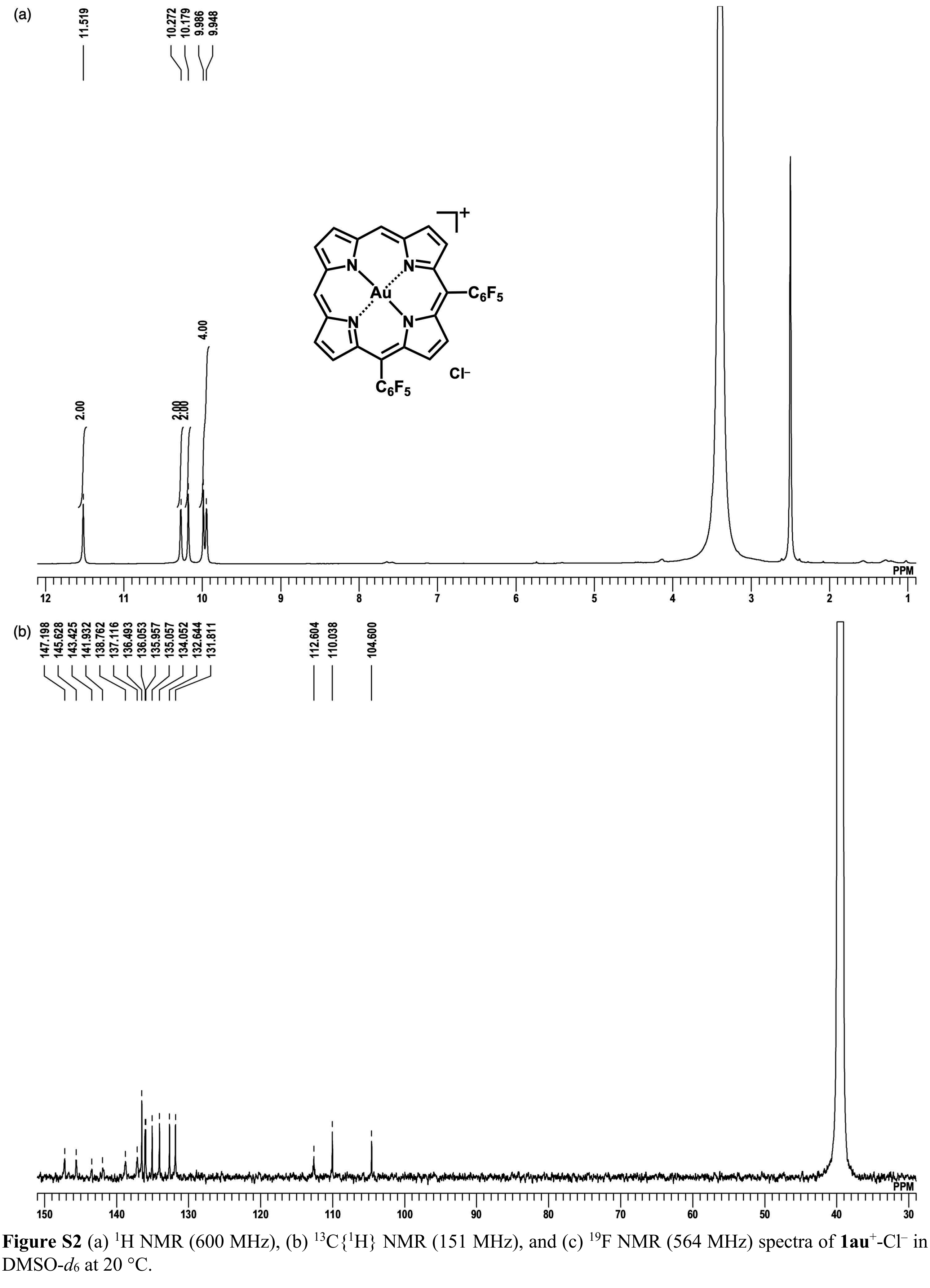

Figure S2 (a) ^{1}H NMR (600 MHz), (b) ^{13}C{^{1}H} NMR (151 MHz), and (c) ^{19}F NMR (564 MHz) spectra of **1au**$^{+}$-Cl^{-} in DMSO-d_6 at 20 °C.

(c)

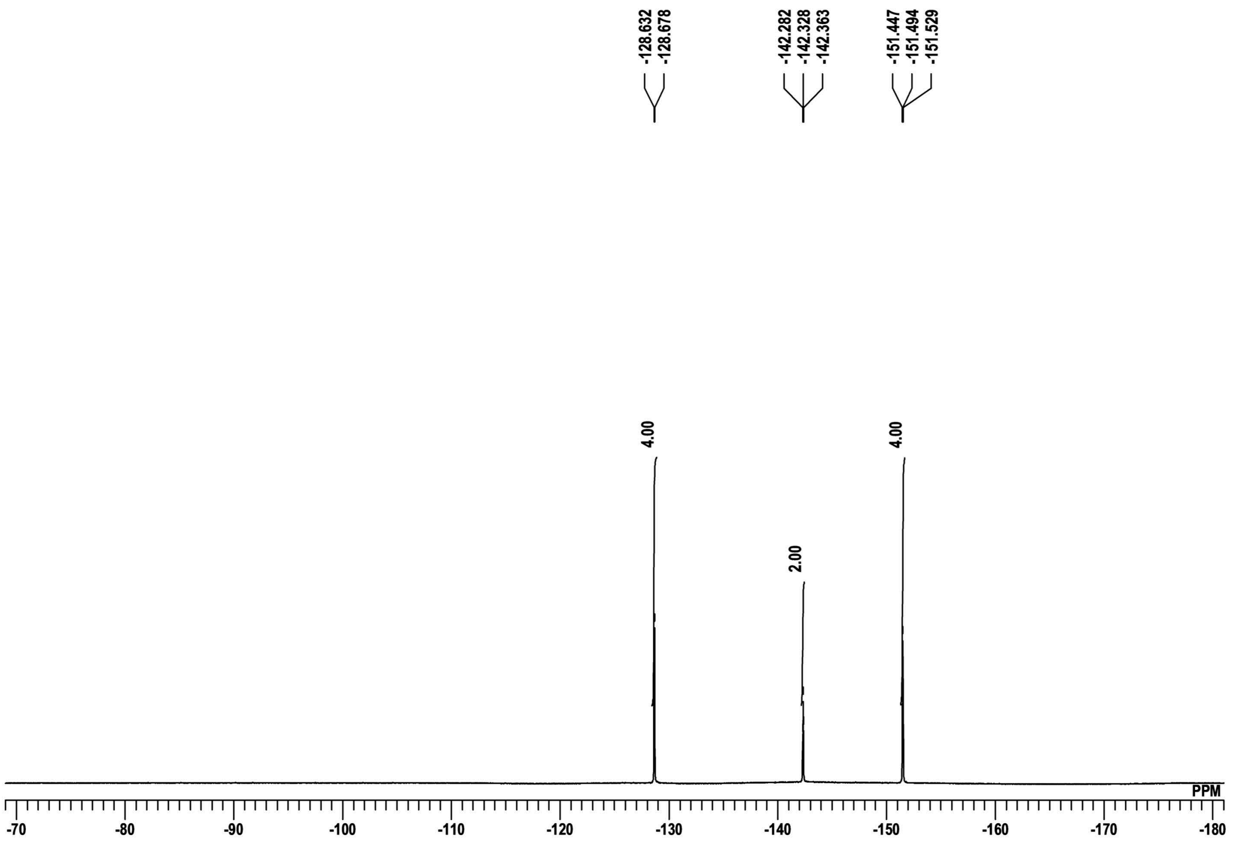

Figure S2 (Continued)

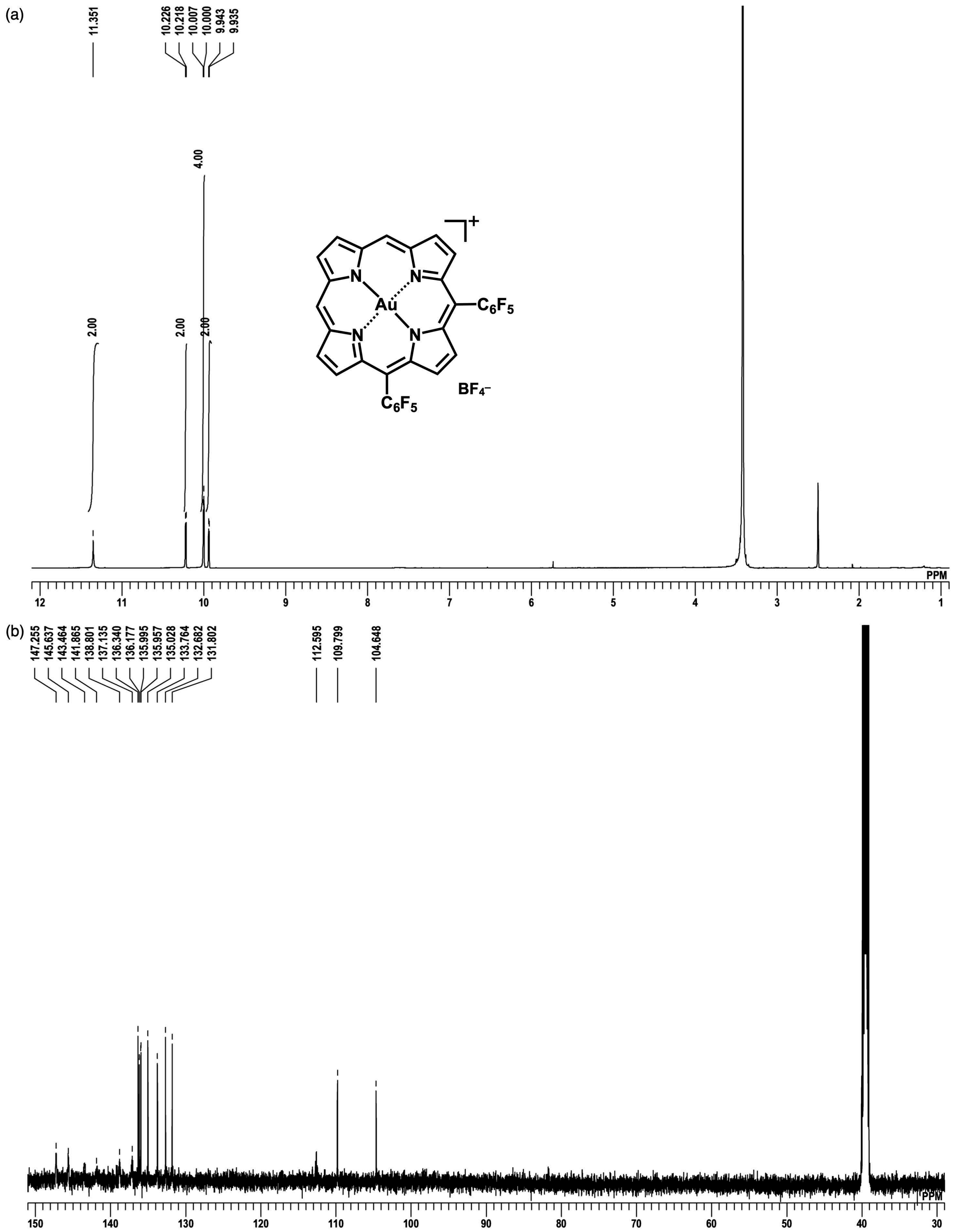

Figure S3 (a) ^{1}H NMR (600 MHz), (b) ^{13}C{^{1}H} NMR (151 MHz), and (c) ^{19}F NMR (564 MHz) of **1au**$^{+}$-BF_4^{-} in DMSO-*d*$_6$ at 20 °C.

(c)

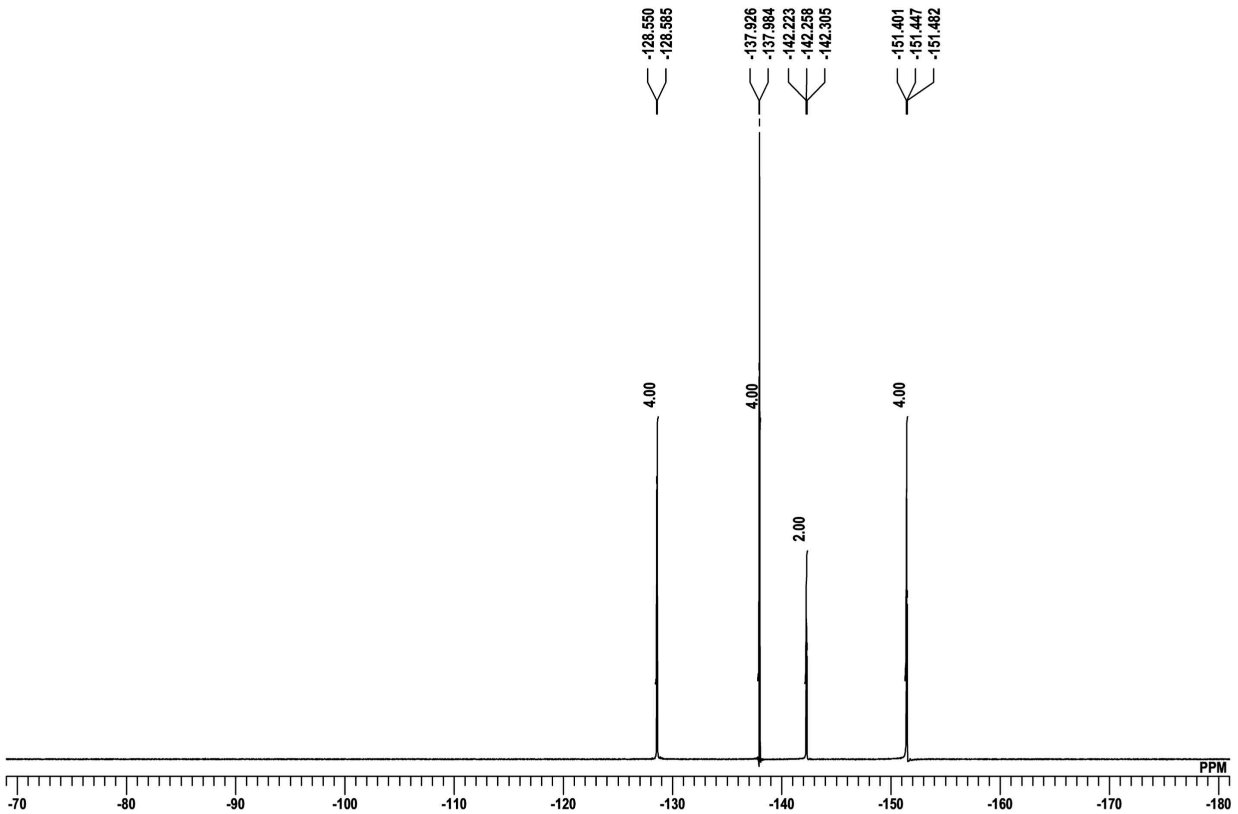

Figure S3 (Continued)

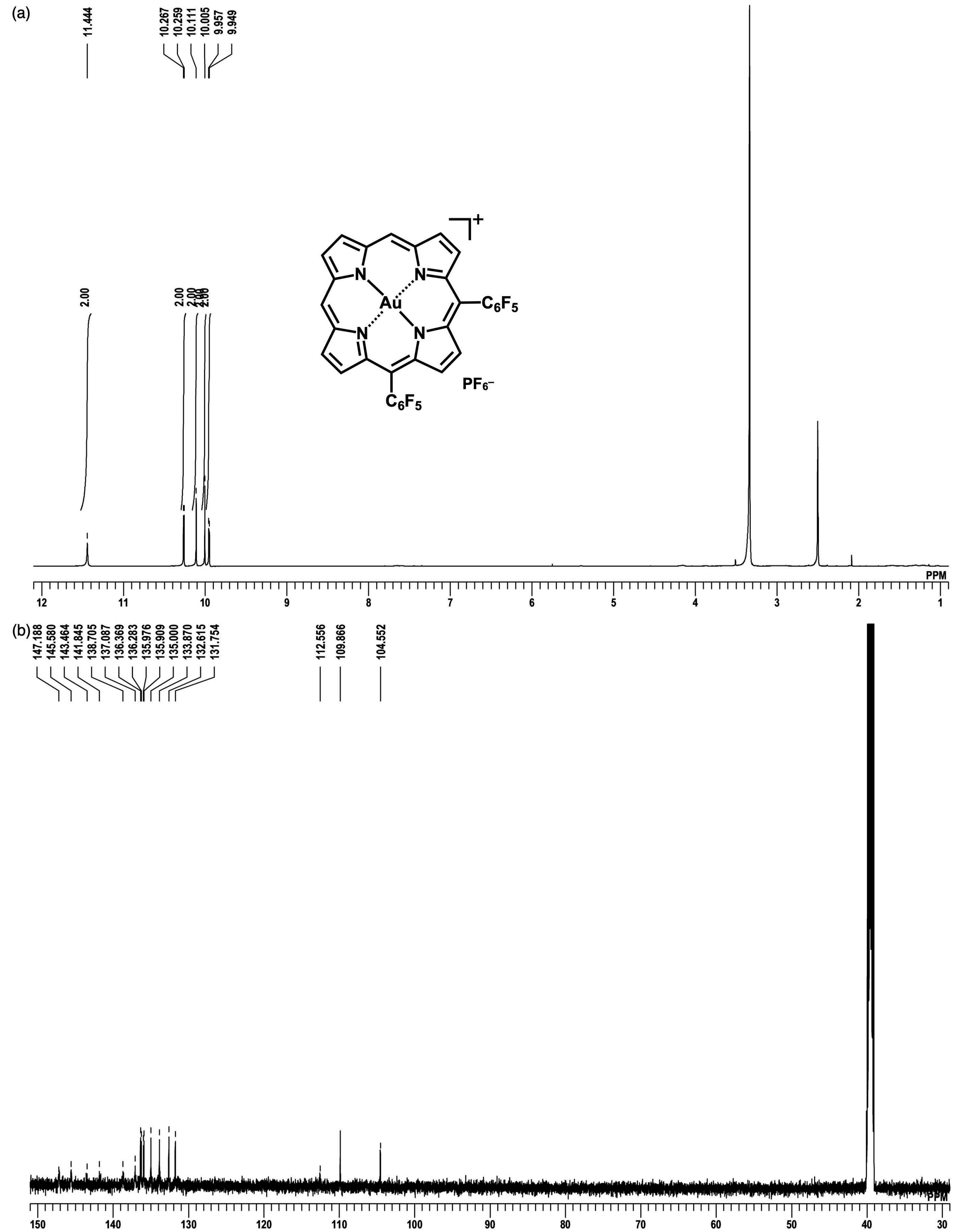

Figure S4 (a) ^{1}H NMR (600 MHz), (b) $^{13}C\{^{1}H\}$ NMR (151 MHz), and (c) ^{19}F NMR (564 MHz) spectra of **1au**$^{+}$-PF_6^{-} in DMSO-d_6 at 20 °C.

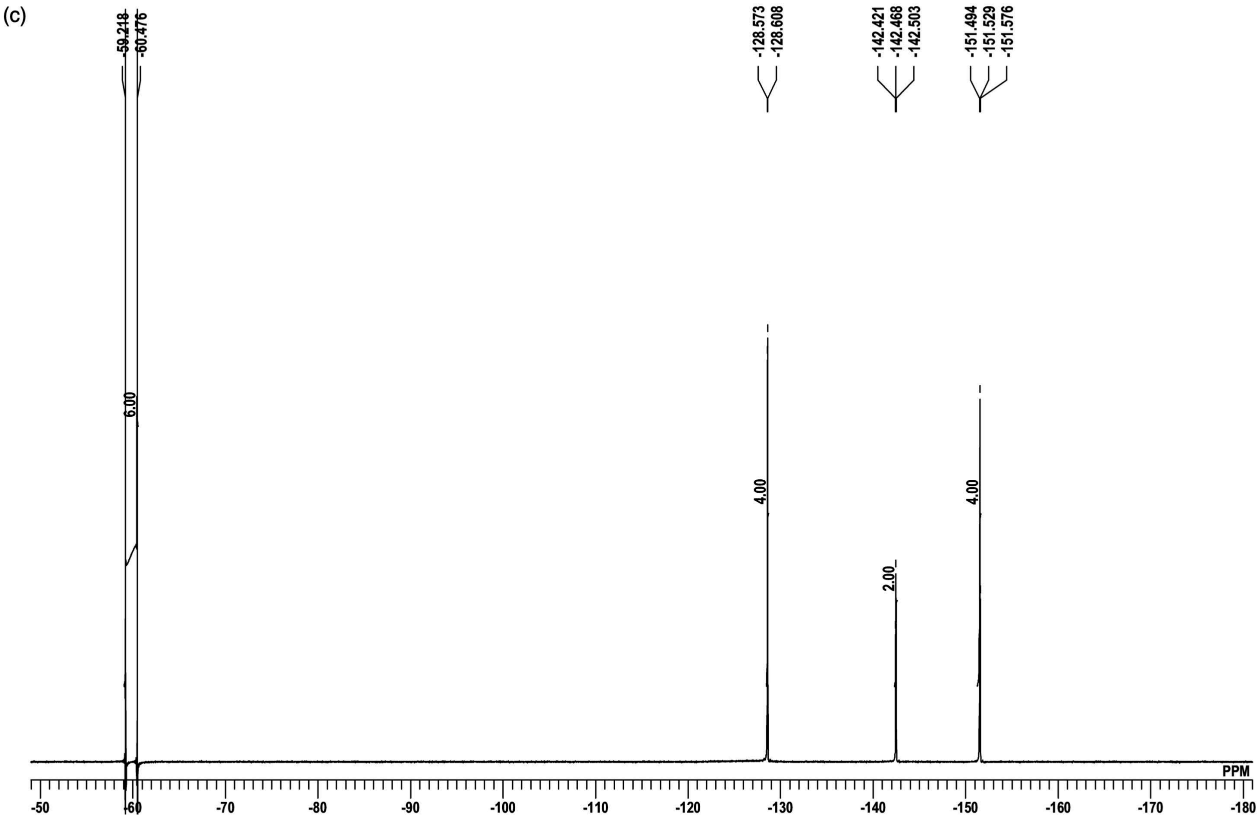

Figure S4 (Continued)

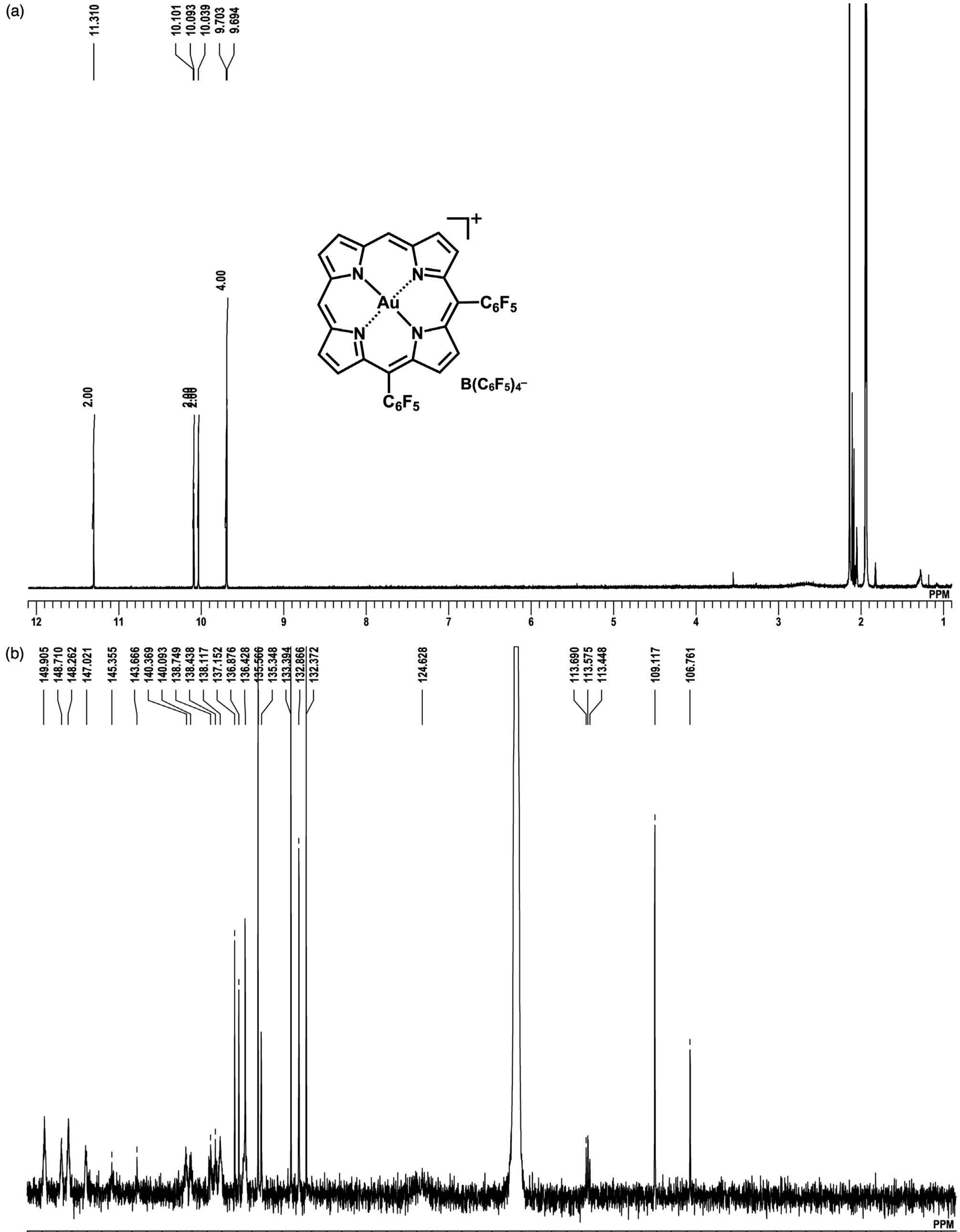

Figure S5 (a) ^{1}H NMR (600 MHz), (b) $^{13}C\{^{1}H\}$ NMR (151 MHz), and (c) ^{19}F NMR (564 MHz) spectra of **1au**$^{+}$-FABA^{-} in CD_3CN at 20 °C.

(c)

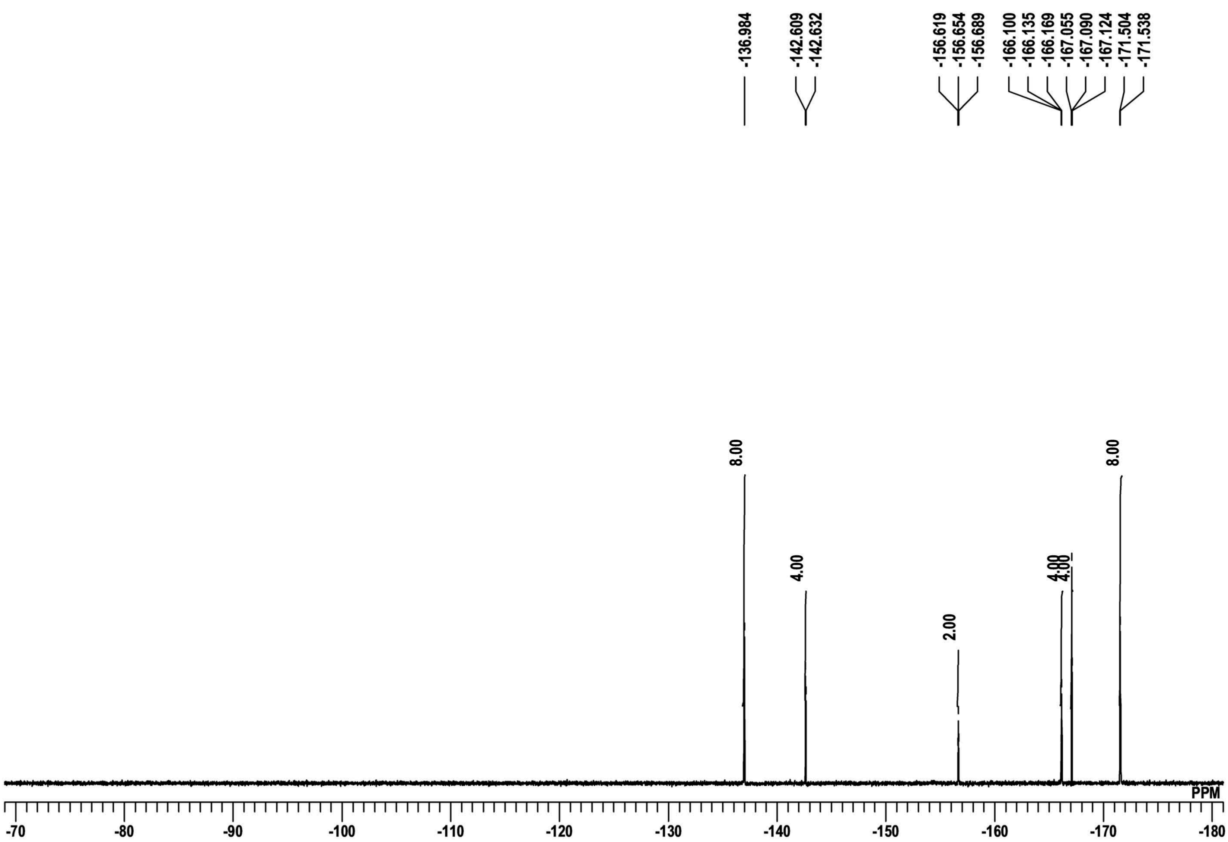

Figure S5 (Continued)

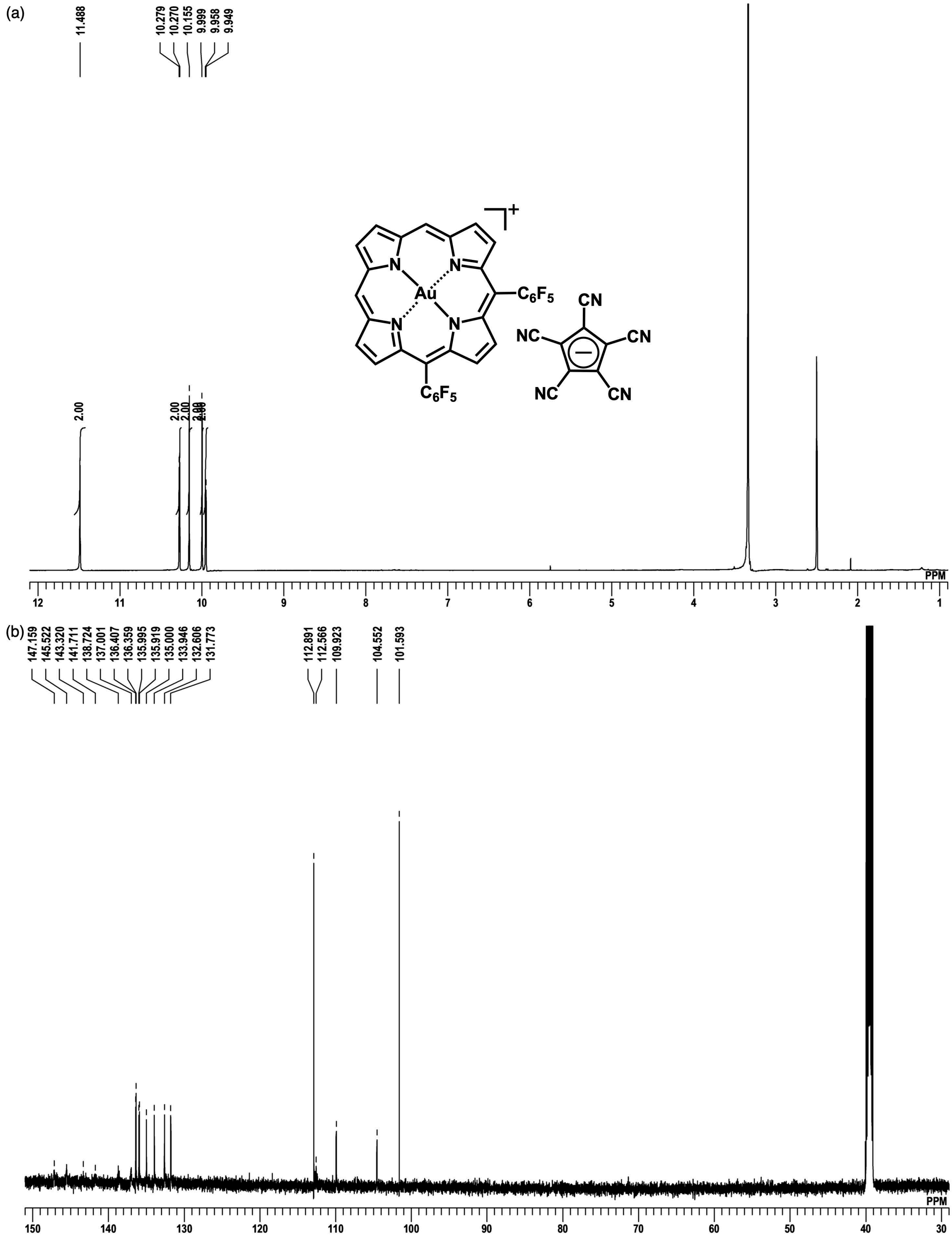

Figure S6 (a) ^{1}H NMR, (b) $^{13}C\{^{1}H\}$ NMR, and (c) ^{19}F NMR spectra of **1au**$^{+}$-PCCp^{-} in DMSO-d_6 at 20 °C.

(c)

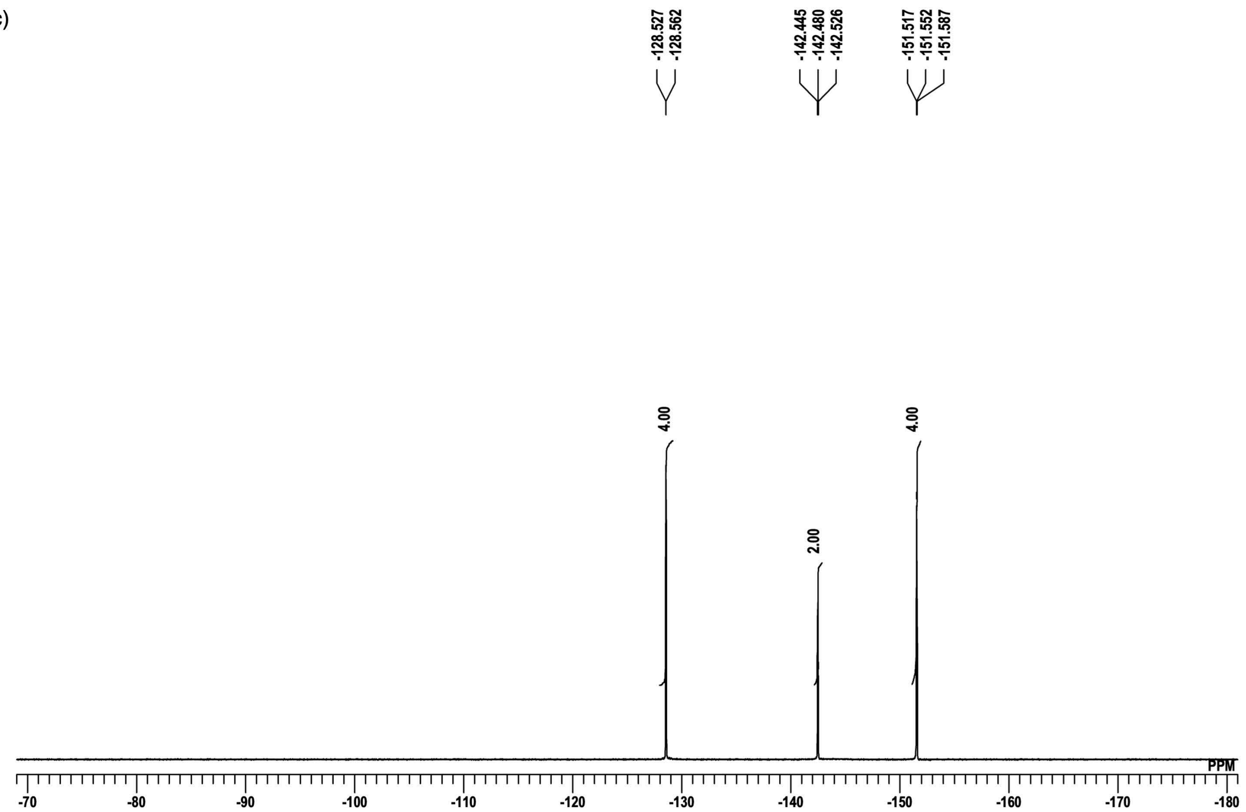

Figure S6 (Continued)

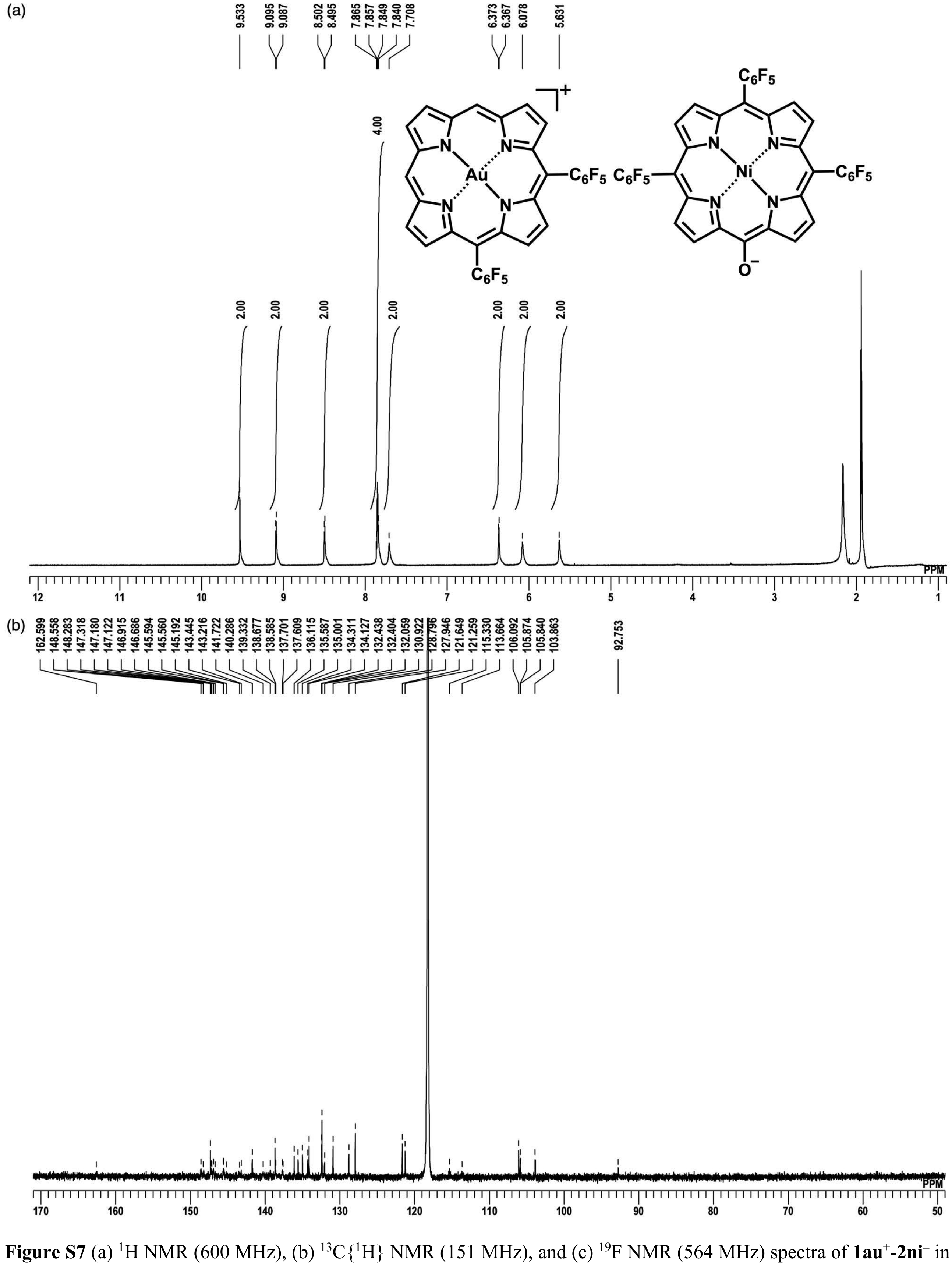

Figure S7 (a) ^{1}H NMR (600 MHz), (b) $^{13}C\{^{1}H\}$ NMR (151 MHz), and (c) ^{19}F NMR (564 MHz) spectra of **1au**$^{+}$-**2ni**$^{-}$ in CD_3CN at 20 °C.

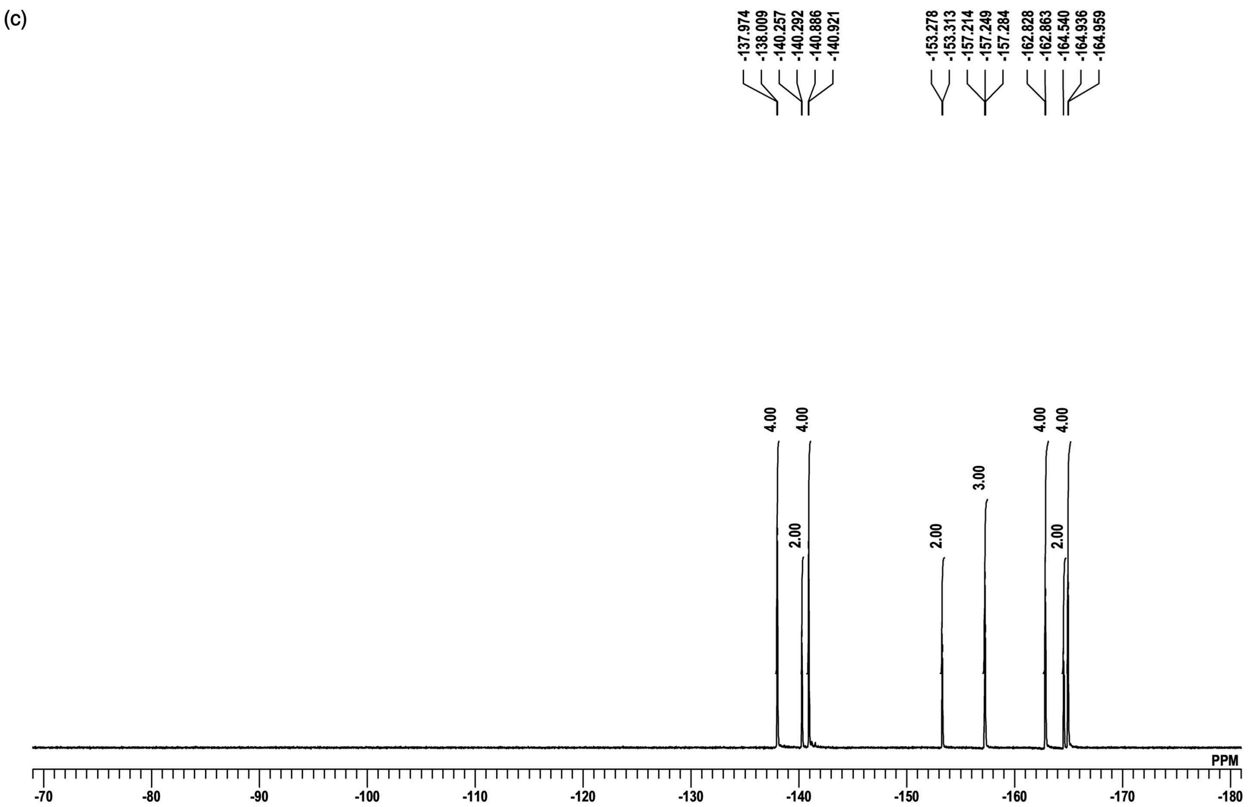

Figure S7 (Continued)

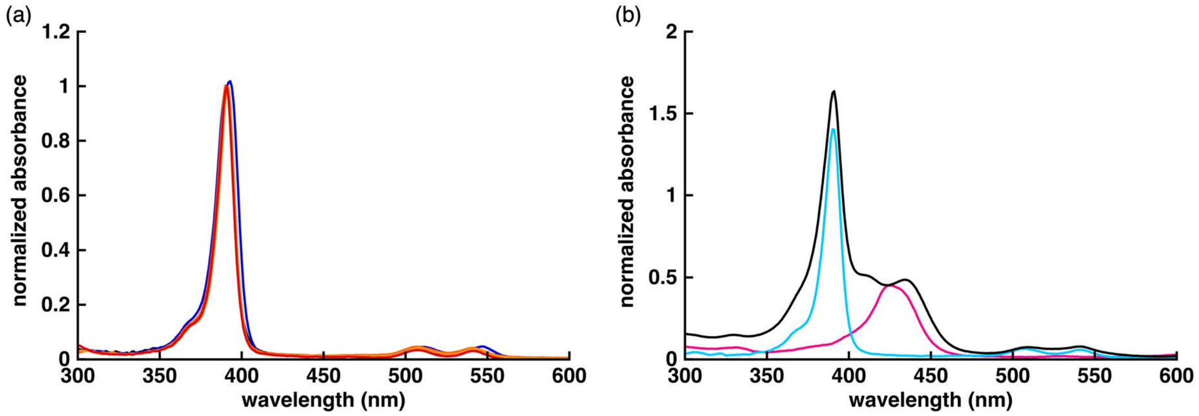

Figure S8 UV/vis absorption spectra in CH_2Cl_2 of (a) **1au**$^+$ as ion pairs with OTf^- (black), Cl^- (blue), PF_6^- (green), $B(C_6F_5)_4^-$ (orange), and $PCCp^-$ (red) and (b) **1au**$^+$-**2ni**$^-$ (black), **1au**$^+$-PF_6^- (cyan), and TBA^+-**2ni**$^-$ [S3b,4b] (magenta). UV/vis absorption spectrum of **1au**$^+$-BF_4^- was excluded in (a) due to the low solubility.

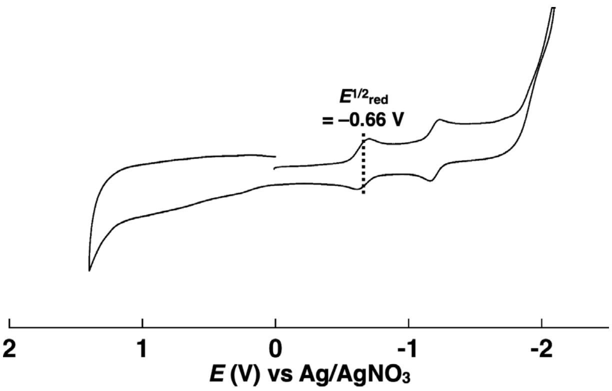

Figure S9 Cyclic voltammogram of **1au**$^+$-PF_6^- in CH_2Cl_2 (0.1 mM) containing $TBAPF_6$ (0.1 M) as an electrolyte under Ar atmosphere at a scan rate of 100 mV s^{-1}. The first reduction potential (versus Ag^+/Ag) of **1au**$^+$ was estimated to be –0.66 V, which was more unlikely to be reduced by 0.11 V than that of 5,10,15-tris(pentafluorophenyl)porphyrin Au^{III} complex **1au′**$^+$.[S4b]

2. X-ray crystallographic data

Method for single-crystal X-ray analysis. Crystallographic data are summarized in Table S1. A single crystal of **1au**$^+$-FABA^-_D was obtained by vapor diffusion of *n*-hexane into a CH_2Cl_2 solution. The data crystal was a red prism of approximate dimensions 0.20 mm × 0.05 mm × 0.05 mm. A single crystal of **1au**$^+$-FABA^-_A was obtained by vapor diffusion of water into an CH_3CN solution. The data crystal was a red prism of approximate dimensions 0.050 mm × 0.020 mm × 0.020 mm. A single crystal of **1au**$^+$-PCCp$^-$ was obtained by vapor diffusion of *n*-hexane into an acetone solution. The data crystal was a red prism of approximate dimensions 0.10 mm × 0.10 mm × 0.03 mm. A single crystal of **1au$^+$-2ni$^-$** was obtained by vapor diffusion of *n*-hexane into a $CHCl_3$/CH_3CN mixed solution. The data crystal was an orange prism of approximate dimensions 0.05 mm × 0.05 mm × 0.03 mm. The data of **1au**$^+$-FABA^-_D and **1au**$^+$-PCCp$^-$ were collected at 90 K on a Bruker D8 Venture diffractometer with MoKα radiation (λ = 0.71073 Å) focused by multilayer confocal mirror, whereas those of **1au**$^+$-FABA^-_A and **1au$^+$-2ni$^-$** were collected at 90 and 100 K, respectively, on a DECTRIS PILATUS3 CdTe 1M diffractometer with Si (311) monochromated synchrotron radiation (λ = 0.4144 and 0.4136 Å, respectively) at BL02B1 (SPring-8).[S5] All the structures were solved by dual-space method. The structures were refined by a full-matrix least-squares method by using a SHELXL 2014[S6] (Yadokari-XG).[S7] In each structure, the non-hydrogen atoms were refined anisotropically. CIF files (CCDC-2430240–2430243) can be obtained free of charge from the Cambridge Crystallographic Data Centre via www.ccdc.cam.ac.uk/data_request/cif.

Table S1 Crystallographic details.

	**1au**$^+$-FABA^-_D	**1au**$^+$-FABA^-_A	**1au**$^+$-PCCp$^-$	**1au$^+$-2ni$^-$**
formula	$C_{32}H_{10}AuF_{10}N_4\cdot C_{24}BF_{20}\cdot 2CH_2Cl_2$	$C_{32}H_{10}AuF_{10}N_4\cdot C_{24}BF_{20}\cdot C_2H_3N$	$C_{32}H_{10}AuF_{10}N_4\cdot C_{10}N_5\cdot 2.5C_3H_6O$	$C_{32}H_{10}AuF_{10}N_4\cdot C_{38}H_8F_{15}N_4NiO\cdot 2CHCl_3\cdot 0.5C_2H_3N$
fw	1686.31	1557.51	1172.75	1976.86
crystal size, mm	0.20 × 0.05 × 0.05	0.050 × 0.020 × 0.020	0.10 × 0.10 × 0.03	0.050 × 0.050 × 0.030
crystal system	triclinic	triclinic	monoclinic	monoclinic
space group	*P*-1 (no. 2)	*P*-1 (no. 2)	$P2_1/c$ (no. 14)	*C*2/*c* (no. 15)
a, Å	13.659(2)	12.0872(6)	13.4847(10)	49.32(2)
b, Å	17.965(4)	14.4763(8)	20.231(2)	14.385(6)
c, Å	33.930(7)	15.7752(8)	33.014(3)	38.632(17)
α, °	88.734(7)	77.618(5)	90	90
β, °	83.479(6)	71.758(5)	97.336(3)	100.439(7)
γ, °	83.799(6)	76.660(5)	90	90
V, Å^3	8223(3)	2520.5(2)	8932.6(14)	26957(21)
ρ_{calcd}, gcm^{-3}	2.043	2.052	1.744	1.948
Z	6	2	8	16
T, K	90(2)	90(2)	90(2)	100(2)
μ, mm^{-1}	3.029 ^{a}	0.774 ^{b}	3.391 ^{a}	0.689 ^{b}
no. of reflns	148344	75454	161180	392486
no. of unique reflns	32168	11569	15737	30900
variables	2757	857	1291	2100
λ, Å	0.71073 ^{a}	0.4144 ^{b}	0.71073 ^{a}	0.4136 ^{b}
R_1 ($I > 2\sigma(I)$)	0.0643	0.0172	0.0306	0.1001
wR_2 ($I > 2\sigma(I)$)	0.1402	0.0436	0.0688	0.2254
GOF	1.035	1.034	1.023	1.190

^{a} MoKα radiation. ^{b} Synchrotron radiation.

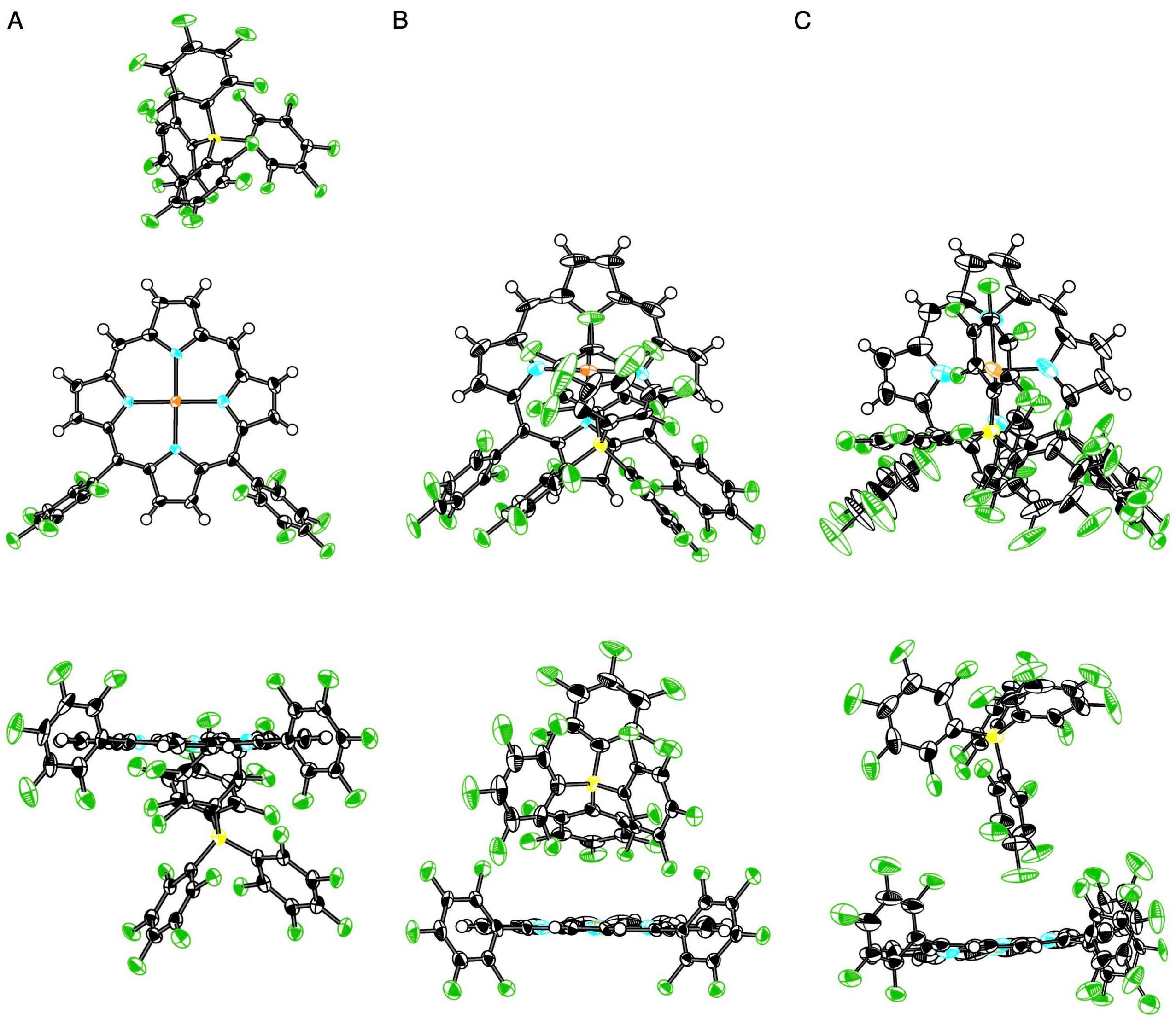

Figure S10 Ortep drawings of single-crystal X-ray structures (top and side views) of **1au**$^{+}$**-**FABA^{-}$_{D}$ as three independent structures (A–C). A pentafluorophenyl group of **1au**$^{+}$ has disordered structures in the ratio of 69 (gray bond) : 32 (white bond). Thermal ellipsoids are scaled to the 50% probability level. Solvent molecules are omitted for clarity. Atom color code: black, white (sphere), yellow, blue, green, and orange refer to carbon, hydrogen, boron, nitrogen, fluorine, and gold, respectively.

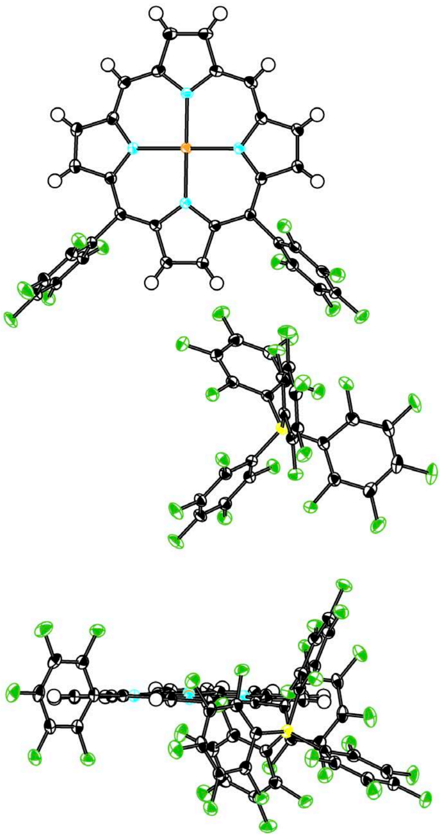

Figure S11 Ortep drawings of single-crystal X-ray structures (top and side views) of **1au**$^+$-FABA^-_A. Thermal ellipsoids are scaled to the 50% probability level. Solvent molecules are omitted for clarity. Atom color code: black, white (sphere), yellow, blue, green, and orange refer to carbon, hydrogen, boron, nitrogen, fluorine, and gold, respectively.

A B

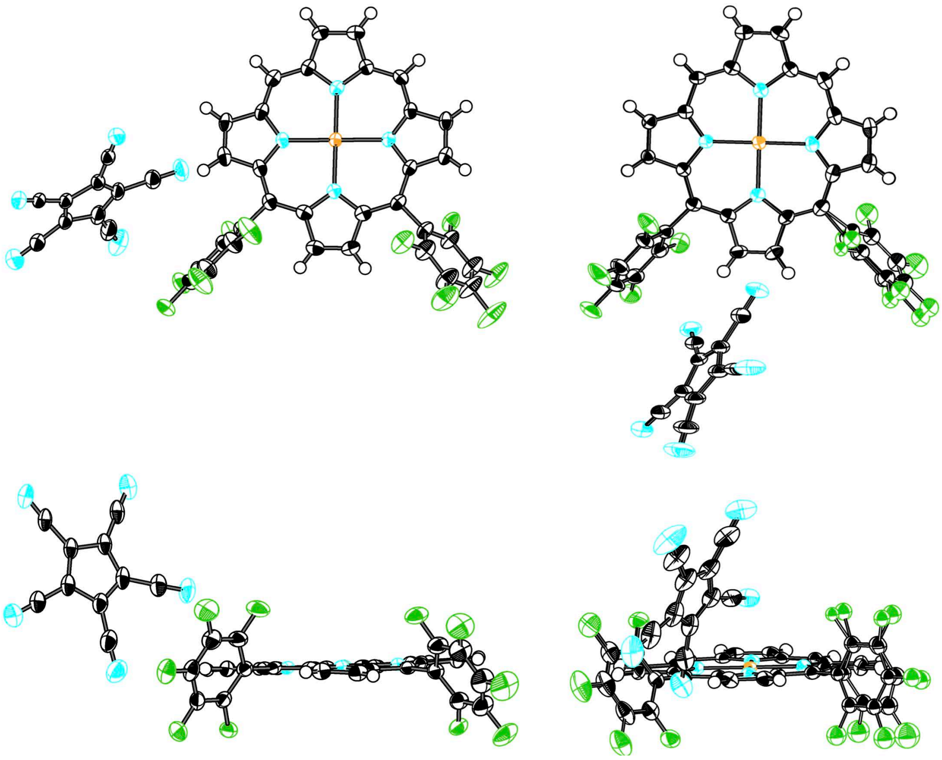

Figure S12 Ortep drawings of single-crystal X-ray structures (top and side views) of **1au**$^+$-PCCp$^-$ as two independent structures (A,B). A pentafluorophenyl group of **1au**$^+$ has disordered structures in the ratio of 69 (gray bond) : 32 (white bond). Thermal ellipsoids are scaled to the 50% probability level. Solvent molecules are omitted for clarity. Atom color code: black, white (sphere), blue, green, and orange refer to carbon, hydrogen, nitrogen, fluorine, and gold, respectively.

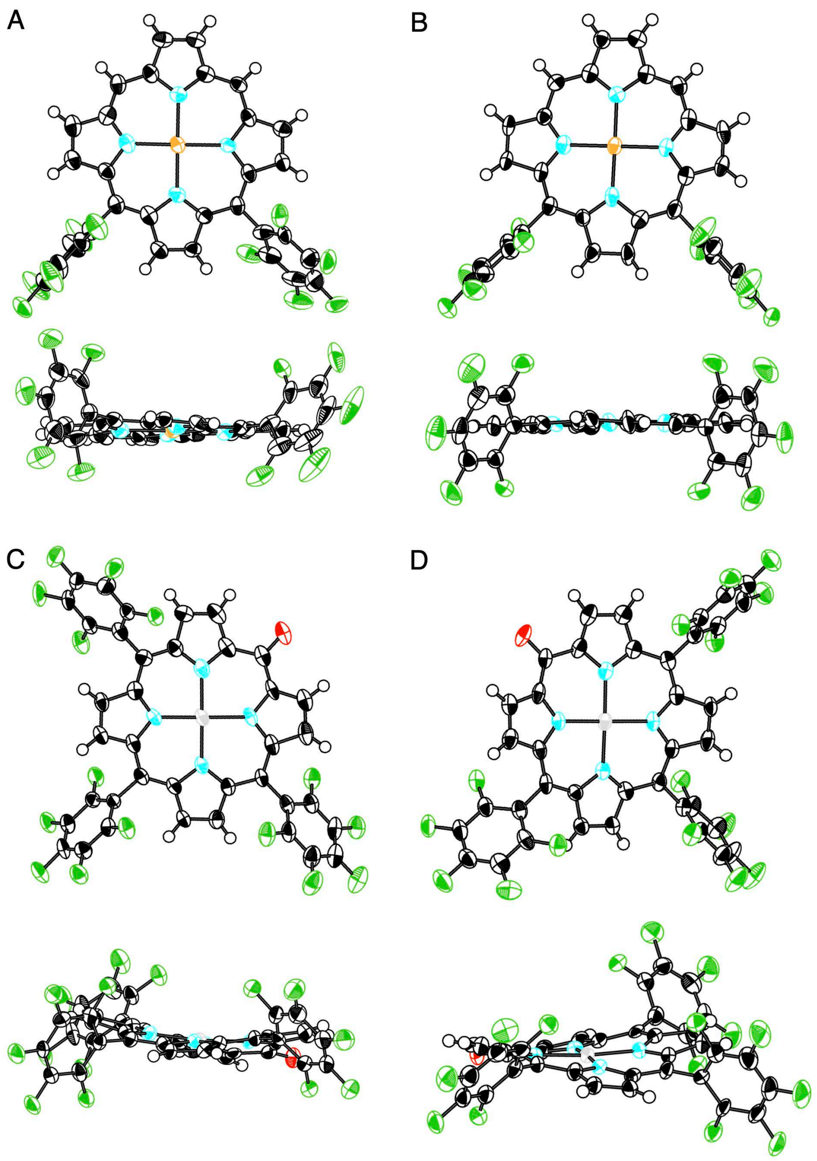

Figure S13 Ortep drawings of single-crystal X-ray structures (top and side views) of **1au**$^{+}$-**2ni**$^{-}$ as two independent structures of **1au**$^{+}$ and **2ni**$^{-}$ (A–D). Thermal ellipsoids are scaled to the 50% probability level. Solvent molecules are omitted for clarity. Atom color code: black, white (sphere), blue, red, green, gray, and orange refer to carbon, hydrogen, nitrogen, oxygen, fluorine, nickel, and gold, respectively.

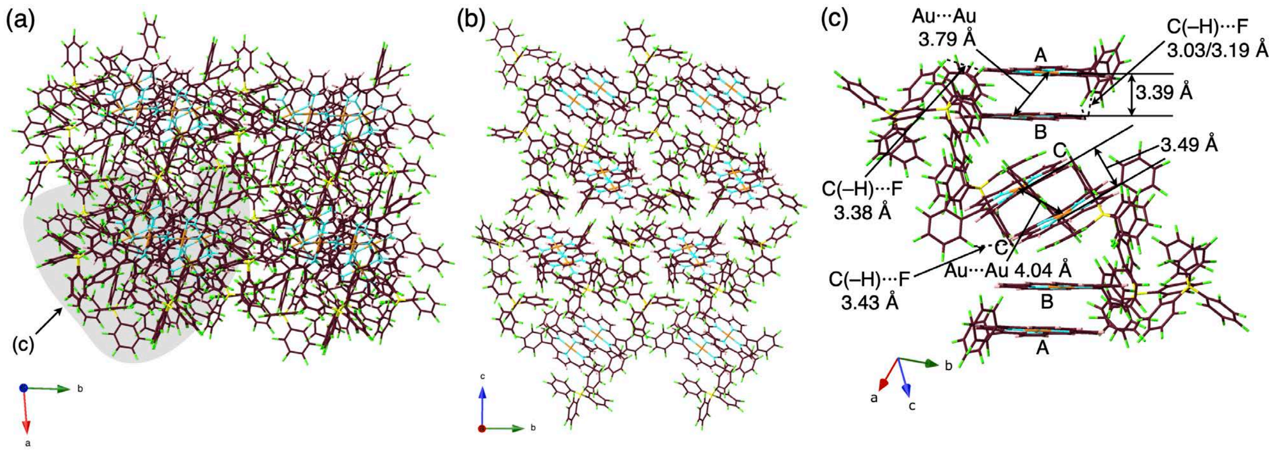

Figure S14 Packing structures of **1au**$^+$-FABA^-_D: (a) top and (b) side views and (c) the stacking model from the arrow shown in (a). The stacking distances between **1au**$^+$ and **1au**$^+$ and Au···Au and C(–H)···F distances in the column are 3.39/3.49, 3.79/4.04, and 3.03–3.43 Å, respectively, suggesting the $^i\pi$–$^i\pi$ and hydrogen-bonding interactions. Color code: brown, pink, yellow, blue, green, and orange refer to carbon, hydrogen, boron, nitrogen, fluorine, and gold, respectively.

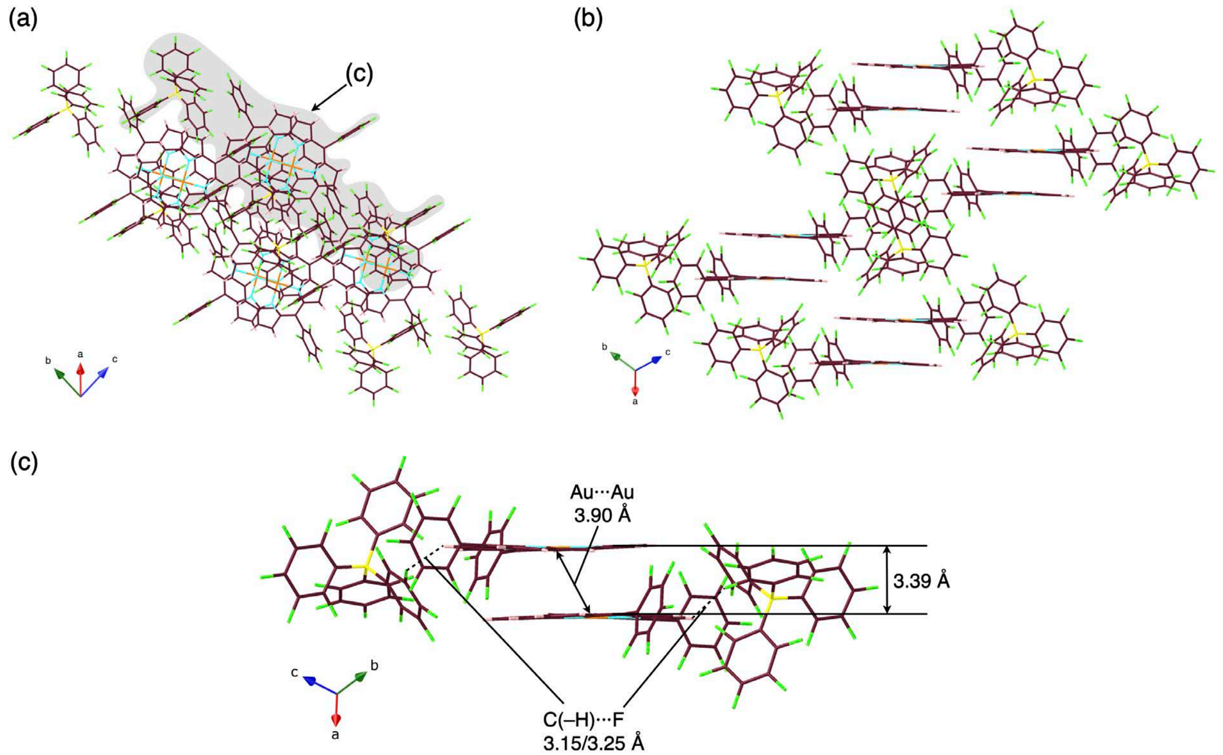

Figure S15 Packing structures of **1au**$^+$-FABA^-_A: (a) top and (b) side views and (c) the stacking model from the arrows shown in (a). The stacking distances between **1au**$^+$ units and Au···Au and C(–H)···F distances in the column are 3.39, 3.90, and 3.15–3.25 Å, respectively, suggesting that the packing structure is stabilized by $^i\pi$–$^i\pi$ and hydrogen-bonding interactions. Atom color code: brown, pink, yellow, blue, green, and orange refer to carbon, hydrogen, boron, nitrogen, fluorine, and gold, respectively.

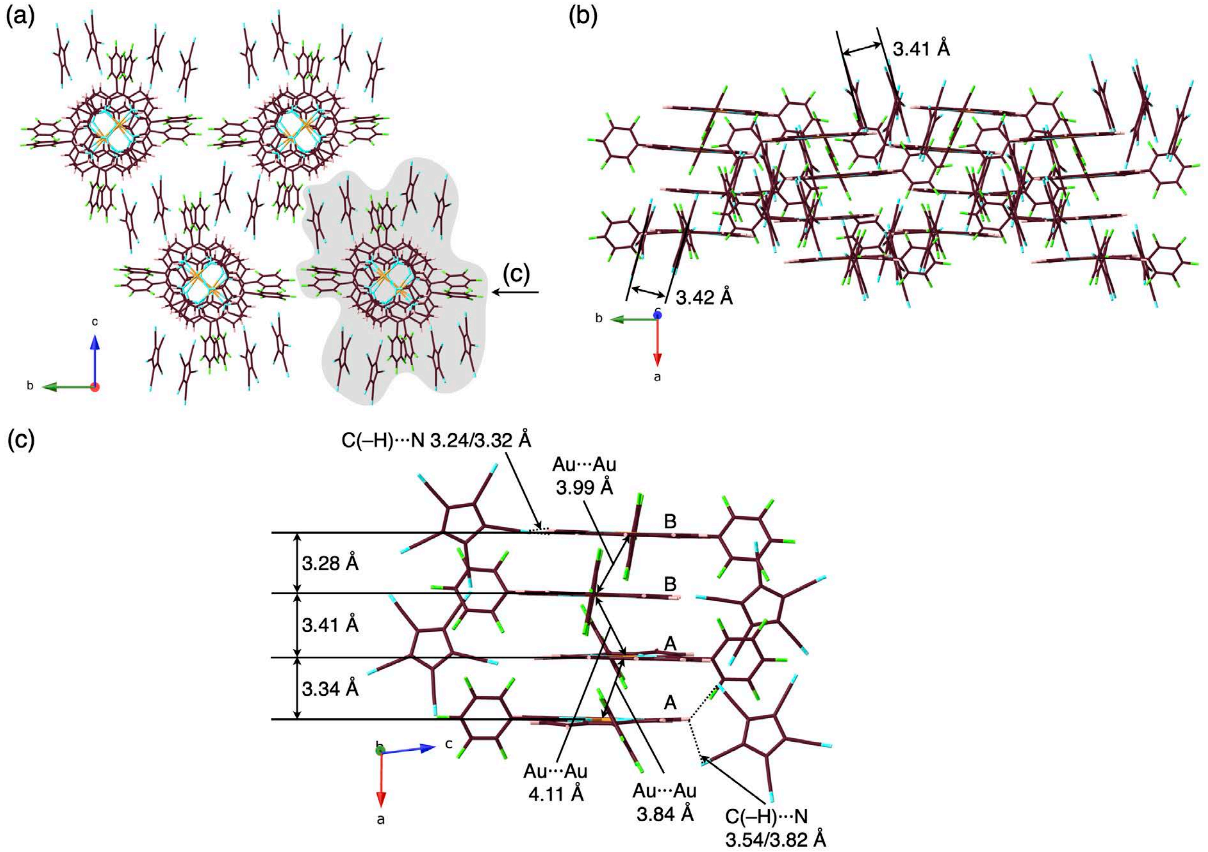

Figure S16 Packing structures of **1au**$^{+}$-PCCp^{-}: (a) top and (b) side views and (c) the stacking model from the arrows shown in (a). The stacking distances between **1au**$^{+}$ units, those between PCCp^{-} units, and Au···Au and C(–H)···N distances in the column are 3.28/3.41/3.34, 3.41/3.42, 3.99/4.11/3.84, and 3.24–3.82 Å, respectively, suggesting that stacking is stabilized by $^{i}\pi$–$^{i}\pi$ and hydrogen-bonding interactions. Atom color code: brown, pink, blue, green, and orange refer to carbon, hydrogen, nitrogen, fluorine, and gold, respectively.

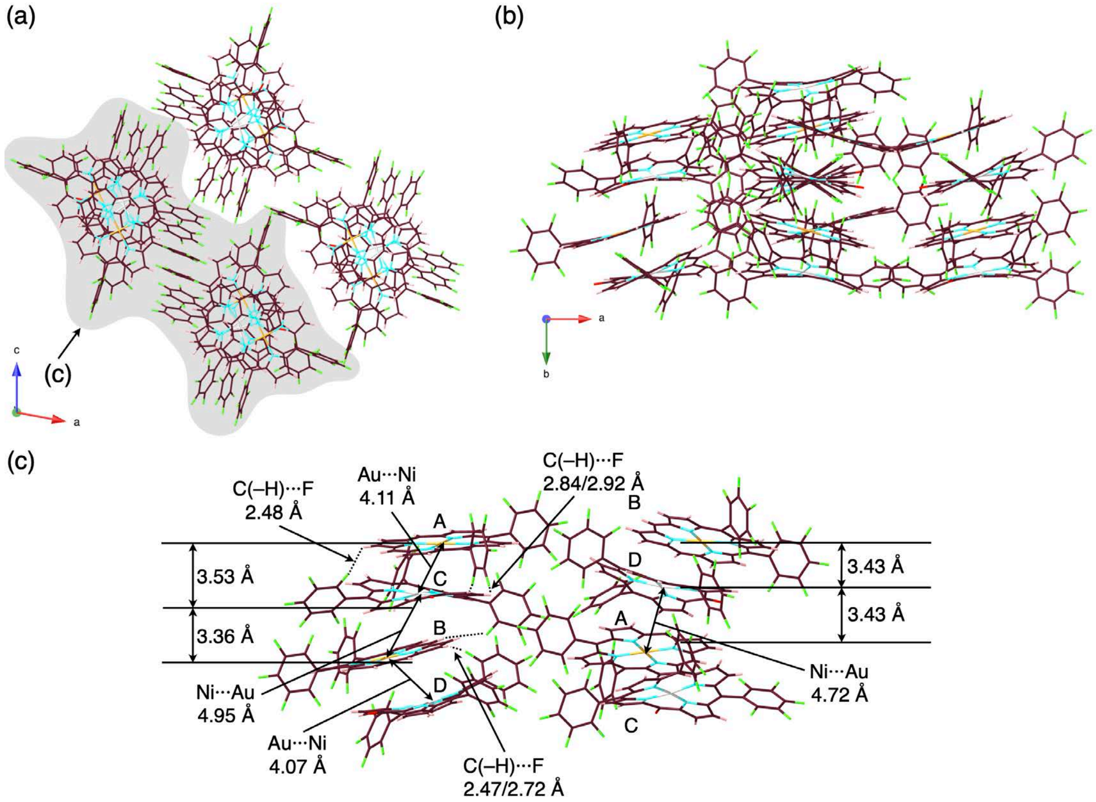

Figure S17 Packing structures of **1au**$^+$-**2ni**$^-$: (a) top and (b) side views and (c) the stacking model from the arrows shown in (a). The stacking distances between **1au**$^+$ and **2ni**$^-$ and Au···Ni and C(–H)···F distances in the column are 3.53/3.36/3.43/3.43, 4.11/4.95/4.08/4.72, and 2.47–2.92 Å, suggesting that stacking is stabilized by $^{i}\pi$–$^{i}\pi$ and hydrogen-bonding interactions. Atom color code: brown, pink, blue, red, green, gray, and orange refer to carbon, hydrogen, nitrogen, oxygen, fluorine, nickel, and gold, respectively.

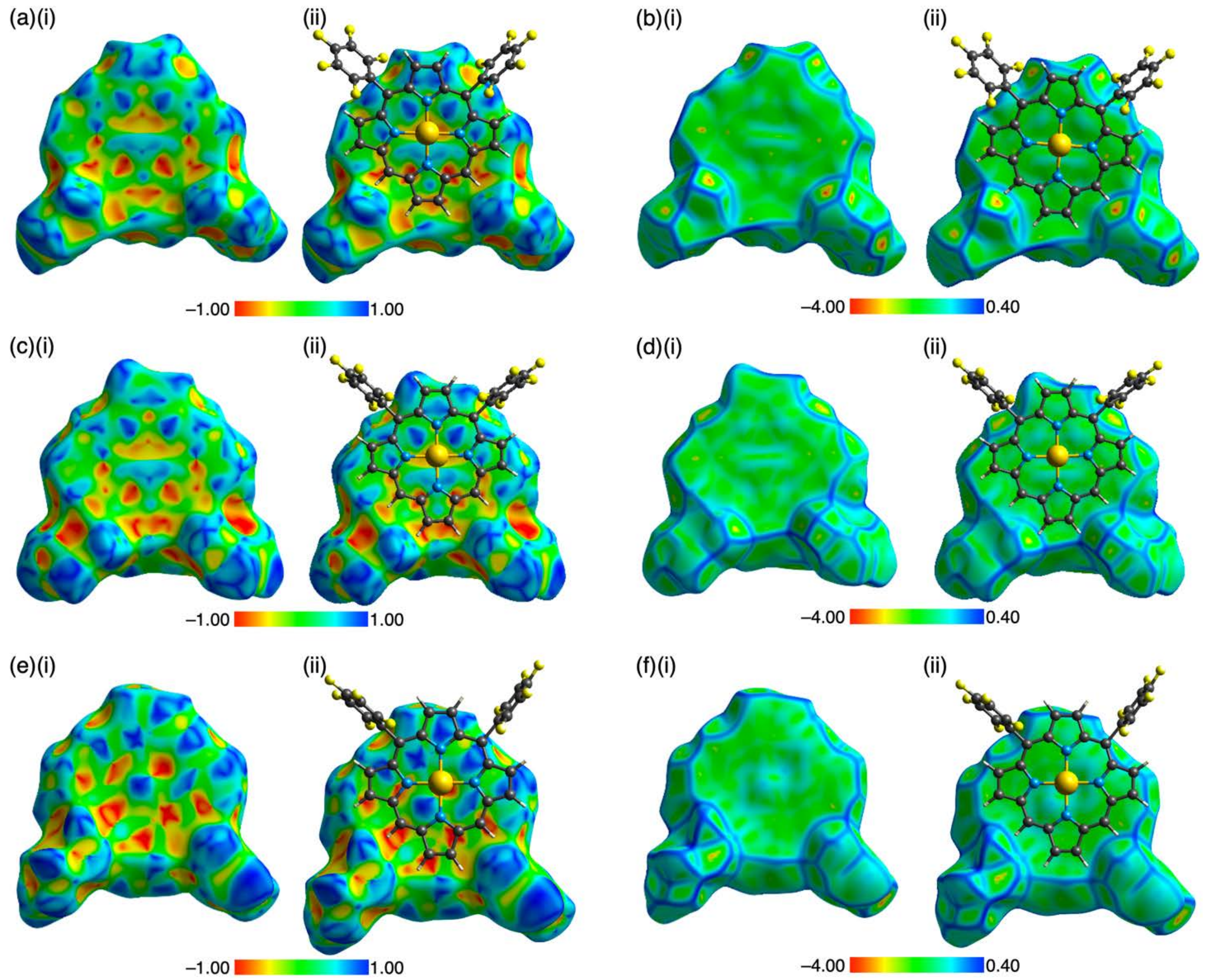

Figure S18 Hirshfeld surface[S8] of **1au**$^{+}$ in the crystal structure of **1au**$^{+}$-FABA$^{-}{}_{D}$ A (a,b), B (c,d), and C (e,f) (a major disordered structure) mapped over (a,c,e) shape-index property and (b,d,f) curvedness property: (i) only surface and (ii) surface with a ball-and-stick model of the neighboring **1au**$^{+}$. Shape index is a qualitative measure of shape and is sensitive to subtle changes in surface shape, particularly in a flat region by differing by sign represent complementary bumps (blue) and hollows (red), whereas curvedness is a function of the root-mean-square curvature of the surface, and maps of curvedness typically show large regions of green (relatively flat) separated by dark blue edges (large positive curvature). The flat region on the curvedness surface suggested the characteristic mapping pattern for stacking in dimeric **1au**$^{+}$. Atom color code: black, pink, blue, yellow, and orange refer to carbon, hydrogen, nitrogen, fluorine, and gold, respectively.

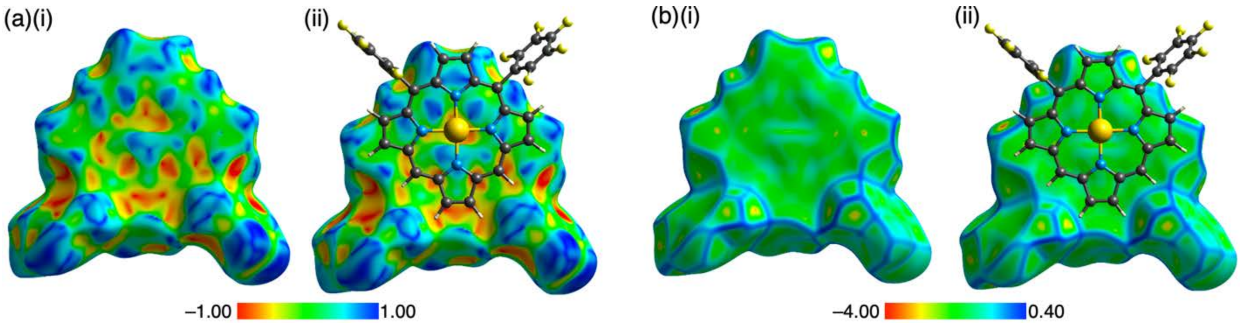

Figure S19 Hirshfeld surface[S8] of **1au**$^{+}$ in the crystal structure of **1au**$^{+}$-FABA$^{-}_{A}$ mapped over (a) shape-index property and (b) curvedness property: (i) only surface and (ii) surface with a ball-and-stick model of the neighboring **1au**$^{+}$. Shape index is a qualitative measure of shape and is sensitive to subtle changes in surface shape, particularly in a flat region by differing by sign represent complementary bumps (blue) and hollows (red), whereas curvedness is a function of the root-mean-square curvature of the surface, and maps of curvedness typically show large regions of green (relatively flat) separated by dark blue edges (large positive curvature). The flat region on the curvedness surface suggested the characteristic mapping pattern for stacking in dimeric **1au**$^{+}$. Atom color code: black, pink, blue, yellow, and orange refer to carbon, hydrogen, nitrogen, fluorine, and gold, respectively.

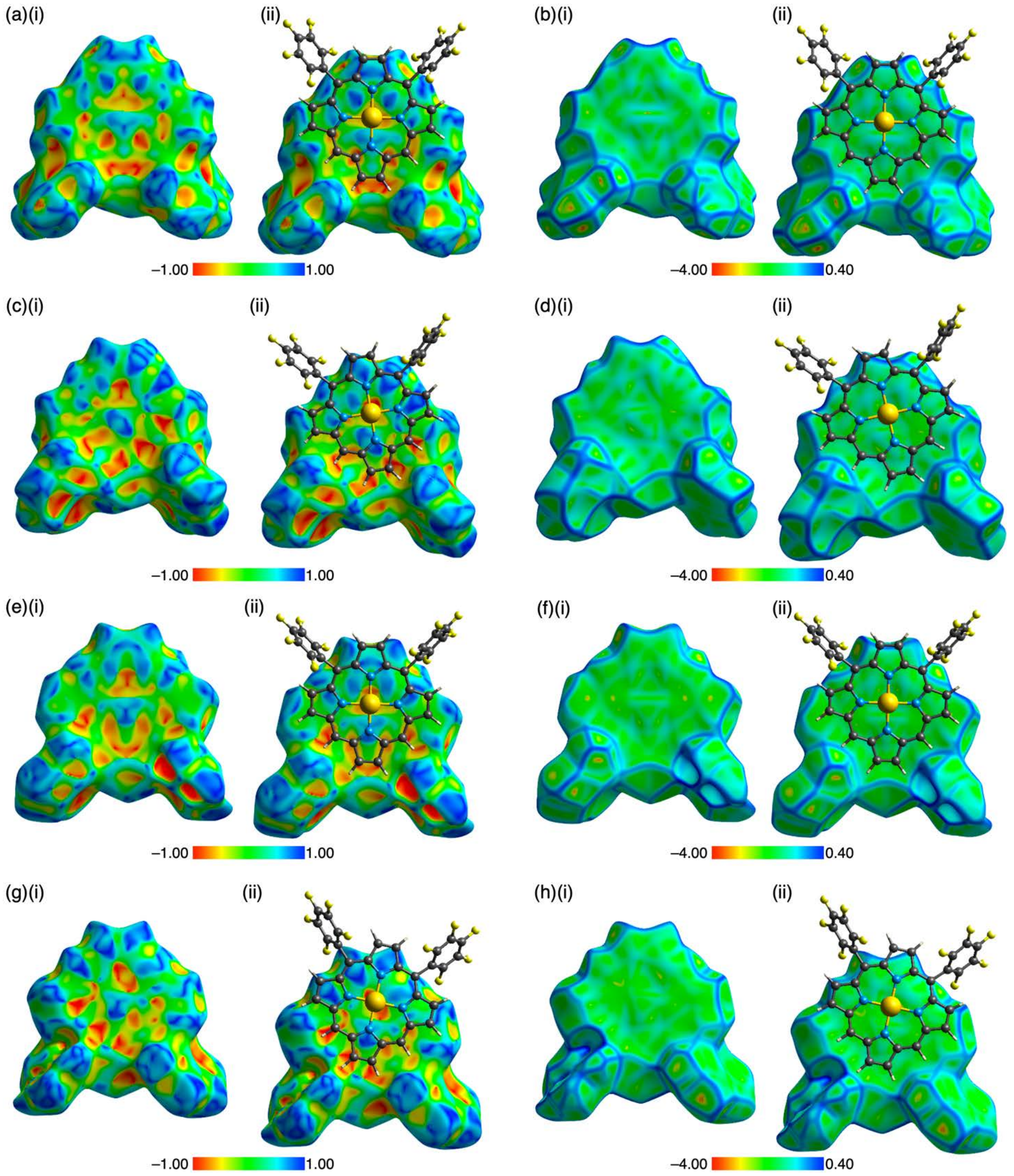

Figure S20 Hirshfeld surface[S8] of **1au**$^+$ in the crystal structure of **1au**$^+$-PCCp$^-$ A (a–d) and B (e–h) (a major disordered structure) mapped over (a,c,e,g) shape-index property and (b,d,f,h) curvedness property: (i) only surface and (ii) surface with a ball-and-stick model of the neighboring **1au**$^+$. Shape index is a qualitative measure of shape and is sensitive to subtle changes in surface shape, particularly in a flat region by differing by sign represent complementary bumps (blue) and hollows (red), whereas curvedness is a function of the root-mean-square curvature of the surface, and maps of curvedness typically show large regions of green (relatively flat) separated by dark blue edges (large positive curvature). The flat region on the curvedness surface suggested the characteristic mapping pattern for stacking in dimeric **1au**$^+$. Atom color code: black, pink, blue, yellow, and orange refer to carbon, hydrogen, nitrogen, fluorine, and gold, respectively.

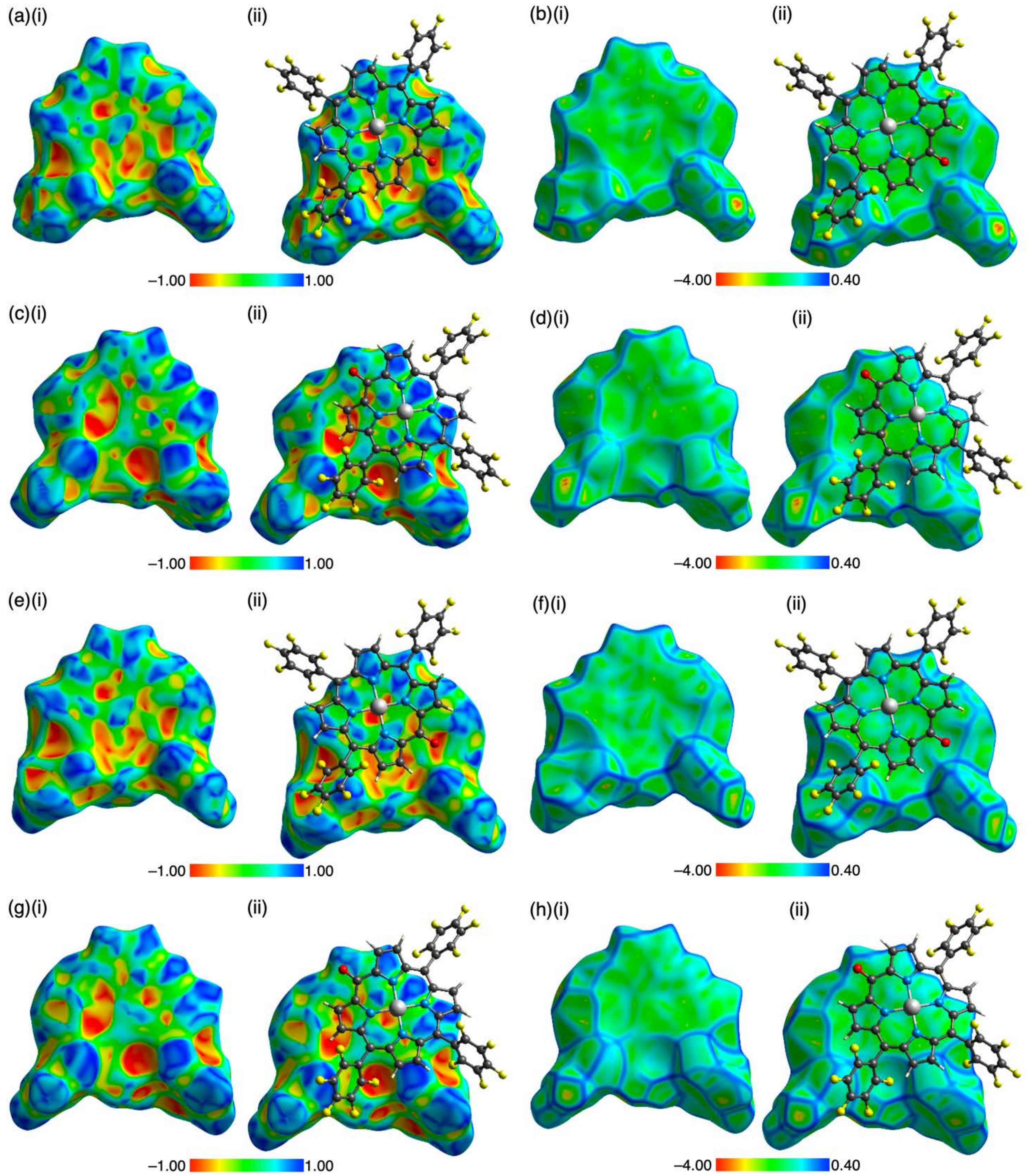

Figure S21 Hirshfeld surface[S8] of **1au**$^+$ in the crystal structure of **1au**$^+$-**2ni**$^-$ A (a–d) and B (e–h) mapped over (a,c,e,g) shape-index property and (b,d,f,h) curvedness property: (i) only surface and (ii) surface with a ball-and-stick model of the neighboring **1au**$^+$ and **2ni**$^-$. Shape index is a qualitative measure of shape and is sensitive to subtle changes in surface shape, particularly in a flat region by differing by sign represent complementary bumps (blue) and hollows (red), whereas curvedness is a function of the root-mean-square curvature of the surface, and maps of curvedness typically show large regions of green (relatively flat) separated by dark blue edges (large positive curvature). The flat region on the curvedness surface suggested the characteristic mapping pattern for stacking in dimeric **1au**$^+$ and **2ni**$^-$. Atom color code: black, pink, blue, red, yellow, and white refer to carbon, hydrogen, nitrogen, oxygen, fluorine, and nickel, respectively.

[S5] K. Sugimoto, H. Ohsumi, S. Aoyagi, E. Nishibori, C. Moriyoshi, Y. Kuroiwa, H. Sawa, M. Takata, *AIP Conf. Proc.* **2010**, *1234*, 887–890.
[S6] G. M. Sheldrick, *Acta Crystallogr. Sect. A* **2008**, *64*, 112–122.

[S7] a) K. Wakita, *Yadokari-XG*, *Software for Crystal Structure Analyses*, **2001**; b) C. Kabuto, S. Akine, T. Nemoto, E. Kwon, *J. Cryst. Soc. Jpn.* **2009**, *51*, 218–224.
[S8] P. R. Spackman, M. J. Turner, J. J. McKinnon, S. K. Wolff, D. J. Grimwood, D. Jayatilaka, M. A. Spackman, *J. Appl. Cryst.* **2021**, *54*, 1006–1011.

3. Theoretical studies

DFT calculations. DFT calculations for the geometrical optimizations were carried out by using the *Gaussian 16* program.[S9]

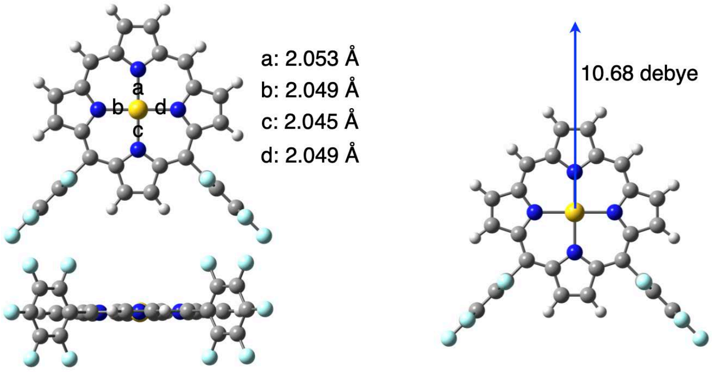

Figure S22 Optimized structure of **1au**$^+$ as top and side views and a top view with a dipole blue arrow at B3LYP/6-+31G(d,p) with LanL2DZ for Au.

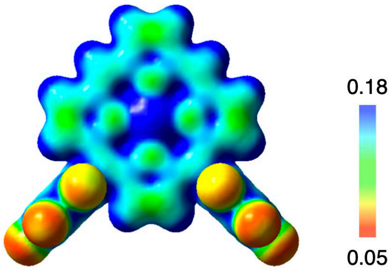

Figure S23 Electrostatic potential (ESP) mapping (δ = 0.01) of **1au**$^+$ at B3LYP/6-31+G(d,p) with LanL2DZ for Au.

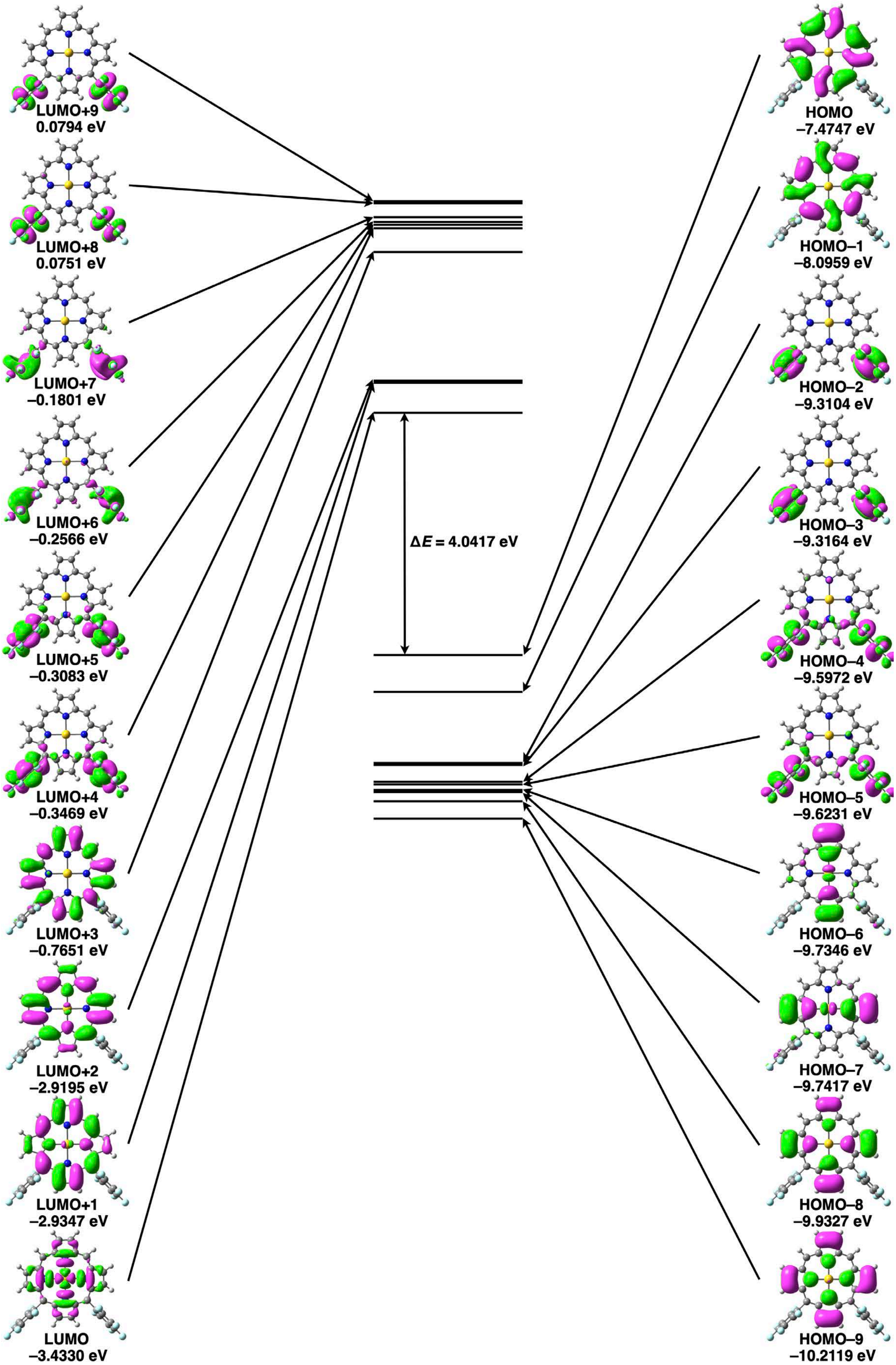

Figure S24 Molecular orbitals (HOMO/LUMO) of **1au**$^+$ estimated at PCM-B3LYP/6-31+G(d,p) with LanL2DZ for Au (CH_2Cl_2) based on the optimized structure at B3LYP/6-31+G(d,p) with LanL2DZ for Au (Figure S22).

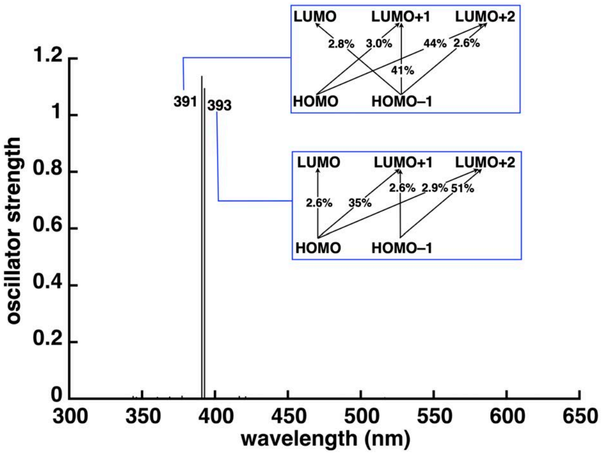

Figure S25 TD-DFT-based UV/vis absorption stick spectra of **1au**$^{+}$ with the transitions correlated with molecular orbitals estimated at PCM-B3LYP/6-31+G(d,p) with LanL2DZ for Au (CH_2Cl_2) based on the optimized structure at B3LYP/6-31+G(d,p) with LanL2DZ for Au (Figure S22).

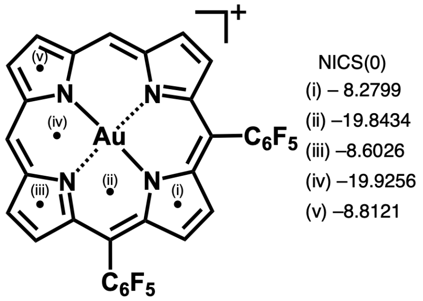

Figure S26 NICS values (ppm)[S10] of **1au**$^{+}$ based on the optimized structure at B3LYP/6-31+G(d,p) with LanL2DZ for Au (Figure S22).

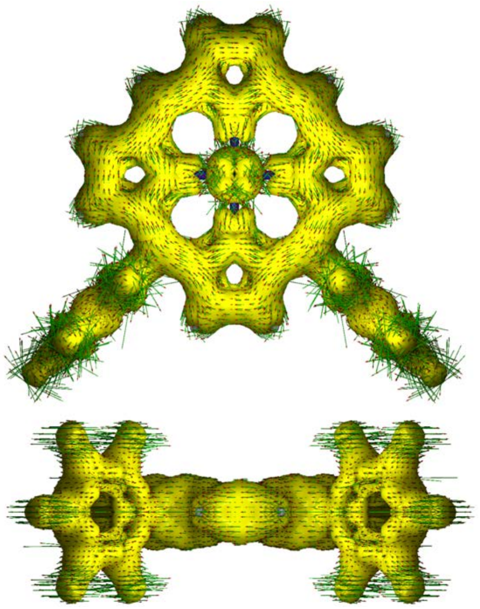

Figure S27 Anisotropy of the induced current density (ACID)[S11] of **1au**$^{+}$ (top and side views) at isosurface value of δ = 0.015 based on the optimized structures (Figure S22) at B3LYP/6-31+G(d,p) with LanL2DZ for Au. The theoretical results were consistent with the NICS values (Figure S26).

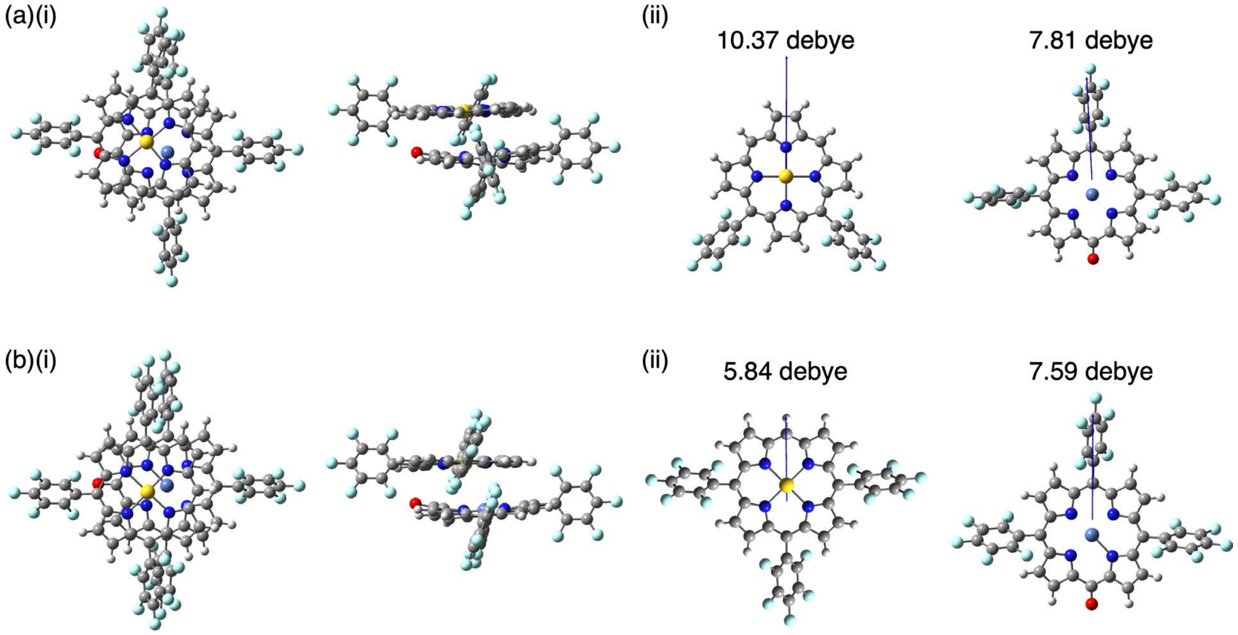

Figure S28 (a) **1au**$^+$-**2ni**$^–$ and (b) **1au**$'^+$-**2ni**$^–$ (**1au**$'^+$: 5,10,15-tris(pentafluorophenyl)porphyrin Au^{III} complex)[S4b] as (i) π-stacked ion pairs (*π-sips*) optimized at GD3BJ-B3LYP/6-31G(d,p) with LanL2DZ for Ni and Au based on the single-crystal X-ray structures and (ii) constituent π-electronic ions (left: cations, right: **2ni**$^–$) with blue arrows representing dipole moments by single-point calculations at B3LYP/6-31+G(d,p) with LanL2DZ for Ni and Au for the optimized structures in the *π-sips*. The larger dipole of **1au**$^+$ in nearly parallel arrangement with that of **2ni**$^–$ and the less electron-withdrawing moieties in **1au**$^+$ would induce the less effective *π-sip* formation for **1au**$^+$-**2ni**$^–$ than **1au**$'^+$-**2ni**$^–$ as seen in Figure S33.

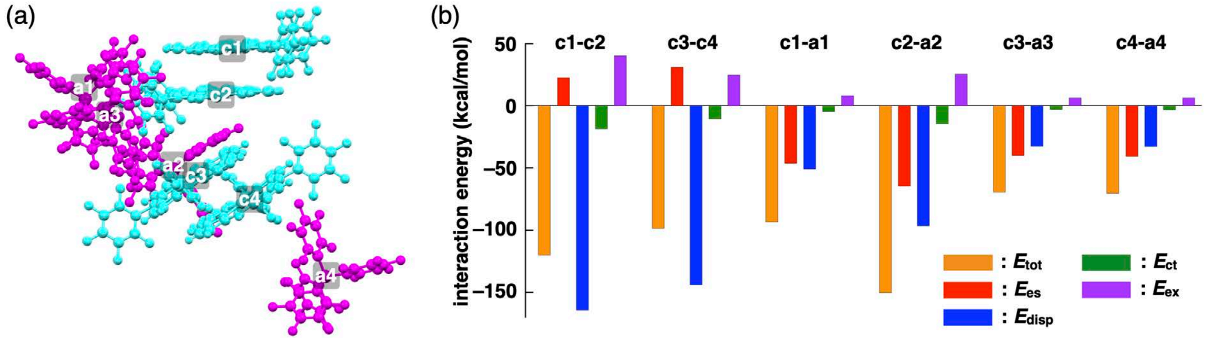

Figure S29 Energy decomposition analysis (EDA)[S12] of **1au**$^+$-FABA$^-_{D}$: (a) single-crystal X-ray structure and (b) intermolecular interaction energies (kcal/mol) between selected ions estimated at an FMO2-MP2 using mixed basis sets including NOSeC-V-DZP with MCP with TZP for Au.[S13–15] The labels (c1–4 and a1–4) correspond to the fragments shown in Table S2.

Table S2 Energies between selected fragments in **1au**$^{+}$-FABA$^{-}_{\mathrm{D}}$ (Figure S29) estimated by EDA calculations[S12] based on an FMO2-MP2 using mixed basis sets including NOSeC-V-DZP with MCP with TZP for Au.[S13–15]

fragments	total interaction energy (E_{tot}) (kcal/mol)	electrostatic interaction energy (E_{es}) (kcal/mol)	dispersion interaction energy (E_{disp}) (kcal/mol)	charge-transfer interaction energy ($E_{\mathrm{ct+mix}}$) (kcal/mol)	exchange repulsion interaction energy (E_{ex}) (kcal/mol)
c1-c2	–119.944	22.453	–164.156	–18.552	40.311
c1-c3	25.090	25.090	0.000	0.000	0.000
c1-c4	19.386	21.169	–1.782	–0.001	0.000
c1-a1	–93.418	–46.304	–50.699	–4.551	8.136
c1-a2	–36.424	–32.189	–4.103	–0.132	0.000
c1-a3	–24.080	–24.080	0.000	0.000	0.000
c1-a4	–16.814	–16.814	0.000	0.000	0.000
c2-c3	24.649	25.937	–1.287	0.000	0.000
c2-c4	22.299	22.299	0.000	0.000	0.000
c2-a1	–43.710	–34.433	–8.839	–0.847	0.409
c2-a2	–150.168	–64.688	–96.556	–14.392	25.468
c2-a3	–47.027	–26.480	–20.944	–1.239	1.635
c2-a4	–68.733	–39.290	–32.821	–2.976	6.354
c3-c4	–98.451	31.093	–144.001	–10.298	24.755
c3-a1	–24.940	–24.940	0.000	0.000	0.000
c3-a2	–73.562	–43.680	–31.005	–2.665	3.788
c3-a3	–69.336	–39.946	–32.736	–2.957	6.302
c3-a4	–25.203	–25.203	0.000	0.000	0.000
c4-a1	–18.920	–18.920	0.000	0.000	0.000
c4-a2	–71.658	–36.604	–37.633	–3.545	6.124
c4-a3	–25.204	–25.204	0.000	0.000	0.000
c4-a4	–70.377	–40.726	–32.811	–3.145	6.304
a1-a2	–5.699	32.080	–43.761	–4.684	10.666
a1-a3	24.903	24.903	0.000	0.000	0.000
a1-a4	17.671	17.671	0.000	0.000	0.000
a2-a3	19.001	31.802	–12.709	–0.217	0.125
a2-a4	26.176	26.176	0.000	0.000	0.000
a3-a4	18.287	18.287	0.000	0.000	0.000

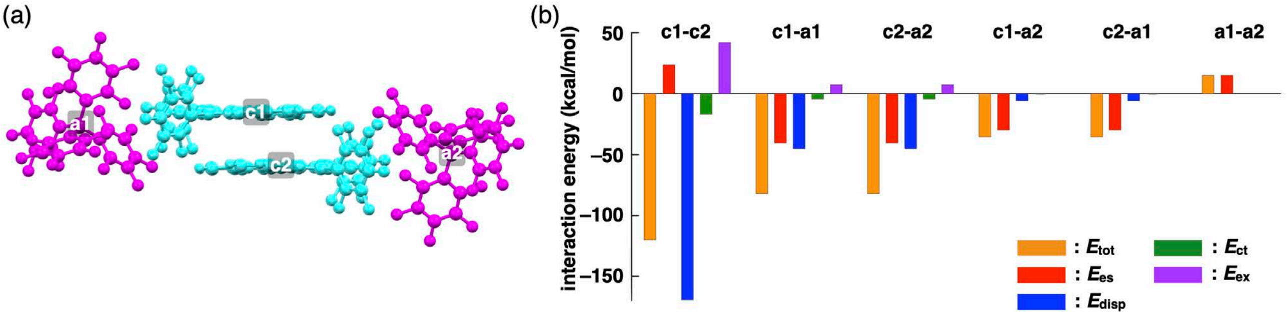

Figure S30 Energy decomposition analysis (EDA)[S12] of **1au**$^{+}$-FABA$^{-}_{\mathrm{A}}$: (a) single-crystal X-ray structure and (b) intermolecular interaction energies (kcal/mol) between selected ions estimated at an FMO2-MP2 using mixed basis sets including NOSeC-V-DZP with MCP with TZP for Au.[S13–15] The labels (c1,2 and a1,2) correspond to the fragments shown in Table S3.

Table S3 Energies between selected fragments in **1au**$^{+}$-FABA$^{-}_{\mathrm{A}}$ (Figure S30) estimated by EDA calculations[S12] based on an FMO2-MP2 using mixed basis sets including NOSeC-V-DZP with MCP with TZP for Au.[S13–15]

fragments	total interaction energy (E_{tot}) (kcal/mol)	electrostatic interaction energy (E_{es}) (kcal/mol)	dispersion interaction energy (E_{disp}) (kcal/mol)	charge-transfer interaction energy ($E_{\mathrm{ct+mix}}$) (kcal/mol)	exchange repulsion interaction energy (E_{ex}) (kcal/mol)
c1-c2	–120.080	23.812	–169.110	–16.864	42.087
c1-a1	–81.971	–40.369	–44.863	–4.166	7.426
c2-a2	–82.000	–40.392	–44.869	–4.168	7.428
c1-a2	–35.591	–29.669	–5.635	–0.427	0.140
c2-a1	–35.585	–29.664	–5.634	–0.427	0.140
a1-a2	14.979	14.979	0.000	0.000	0.000

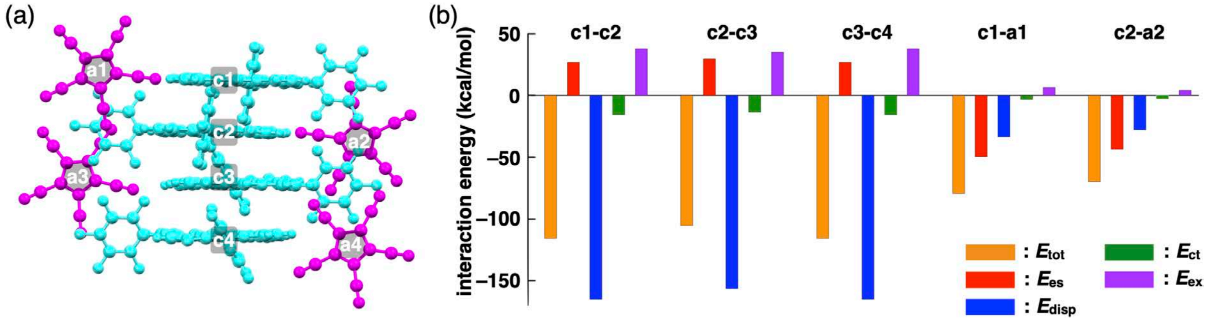

Figure S31 Energy decomposition analysis (EDA)[S12] of **1au**$^+$-PCCp$^-$: (a) single-crystal X-ray structure and (b) intermolecular interaction energies (kcal/mol) between selected ions estimated at an FMO2-MP2 using mixed basis sets including NOSeC-V-DZP with MCP with TZP for Au.[S13–15] The labels (c1–4 and a1–4) correspond to the fragments shown in Table S4.

Table S4 Energies between selected fragments in **1au**$^+$-PCCp$^-$ (Figure S31) estimated by EDA calculations[S12] based on an FMO2-MP2 using mixed basis sets including NOSeC-V-DZP with MCP with TZP for Au.[S13–15]

fragments	total interaction energy (E_{tot}) (kcal/mol)	electrostatic interaction energy (E_{es}) (kcal/mol)	dispersion interaction energy (E_{disp}) (kcal/mol)	charge-transfer interaction energy (E_{ct+mix}) (kcal/mol)	exchange repulsion interaction energy (E_{ex}) (kcal/mol)
c1-c2	–115.625	26.883	–165.075	–15.524	38.091
c1-c3	0.205	33.727	–35.516	–1.561	3.555
c1-c4	27.274	27.274	0.000	0.000	0.000
c1-a1	–79.424	–49.544	–33.351	–3.108	6.579
c1-a2	–52.213	–40.812	–1.734	–1.740	2.073
c1-a3	–30.970	–30.970	0.000	0.000	0.000
c1-a4	–24.755	–22.734	–1.991	–0.031	0.000
c2-c3	–105.193	29.473	–156.221	–13.651	35.206
c2-c4	10.172	36.090	–26.947	–0.628	1.656
c2-a1	–52.221	–40.823	–11.732	–1.739	2.074
c2-a2	–79.455	–49.570	–33.358	–3.110	6.583
c2-a3	–53.183	–41.776	–11.725	–1.755	2.072
c2-a4	–32.623	–30.577	–2.037	–0.009	0.000
c3-c4	–115.625	26.883	–165.075	–15.524	38.091
c3-a1	–29.051	–29.051	0.000	0.000	0.000
c3-a2	–30.970	–30.970	0.000	0.000	0.000
c3-a3	–69.733	–43.493	–27.918	–2.583	4.260
c3-a4	–52.213	–40.812	–11.734	–1.740	2.073
c4-a1	–23.488	–23.488	0.000	0.000	0.000
c4-a2	–32.822	–30.451	–2.359	–0.012	0.000
c4-a3	–52.221	–40.823	–11.732	–1.739	2.074
c4-a4	–61.727	–44.284	–17.706	–1.388	1.651
a1-a2	18.287	18.287	0.000	0.000	0.000
a1-a3	31.098	31.098	0.000	0.000	0.000
a1-a4	17.671	17.671	0.000	0.000	0.000
a2-a3	20.137	20.137	0.000	0.000	0.000
a2-a4	26.176	26.176	0.000	0.000	0.000
a3-a4	18.287	18.287	0.000	0.000	0.000

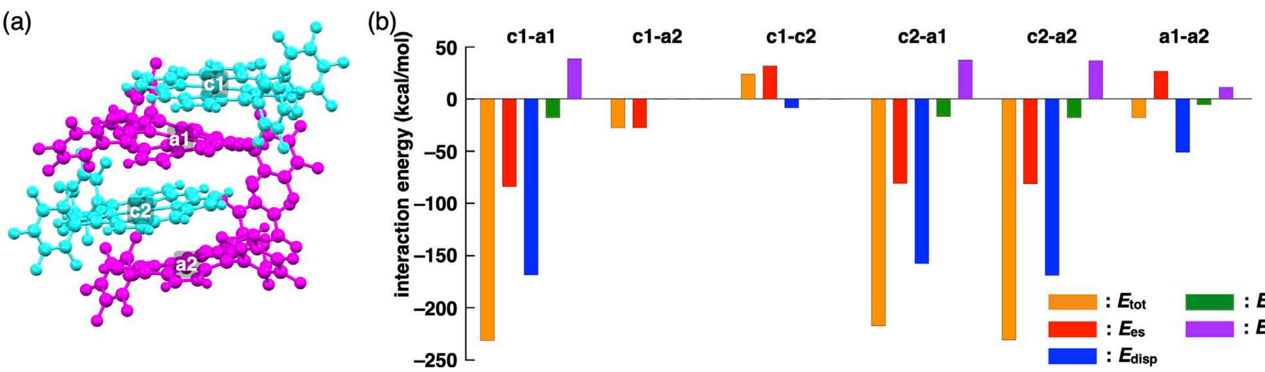

Figure S32 Energy decomposition analysis (EDA)[S12] of **1au**$^+$-**2ni**$^-$: (a) single-crystal X-ray structure and (b) intermolecular interaction energies (kcal/mol) between selected ions estimated at an FMO2-MP2 using mixed basis sets including NOSeC-V-DZP with MCP with TZP for Ni and Au.[S13–15] The labels (c1,2 and a1,2) correspond to the fragments shown in Table S5.

Table S5 Energies between selected fragments in **1au**$^+$-**2ni**$^-$ (Figure S32) estimated by EDA calculations[S12] based on an FMO2-MP2 using mixed basis sets including NOSeC-V-DZP with MCP with TZP for Ni and Au.[S13–15]

fragments	total interaction energy (E_{tot}) (kcal/mol)	electrostatic interaction energy (E_{es}) (kcal/mol)	dispersion interaction energy (E_{disp}) (kcal/mol)	charge-transfer interaction energy (E_{ct+mix}) (kcal/mol)	exchange repulsion interaction energy (E_{ex}) (kcal/mol)
c1-c2	23.930	32.138	–8.196	0.000	–0.012
c1-a1	–231.151	–83.672	–168.133	–17.973	38.628
c1-a2	–27.485	–27.485	0.000	0.000	0.000
c2-a1	–216.995	–80.590	–157.605	–16.629	37.830
c2-a2	–230.729	–81.094	–168.985	–17.660	37.010
a1-a2	–17.866	26.571	–50.929	–5.250	11.741

Cartesian Coordination of 1au$^+$

–2578.0814 hartree

Au,4.5368134108,-0.1997960232,-0.1128213369
C,1.8110078236,-1.5118061503,-0.6134322842
C,1.2051585125,-2.7924386602,-0.850998746
C,2.2009993425,-3.7227381228,-0.8483463139
C,3.4419007273,-3.031726502,-0.6087058431
C,5.8931756703,-2.9465136855,-0.2989709852
C,7.1921041432,-3.5591259531,-0.2191908093
C,8.1001105297,-2.5731510437,0.0177394395
C,7.3837222209,-1.3279826713,0.0899442257
C,7.2683506828,1.1232906185,0.3898828946
C,7.8553085429,2.4170919279,0.6272035865
C,6.8472823944,3.3341591154,0.6217465845
C,5.621116173,2.6254698326,0.3813593497
C,3.1714219735,2.548887828,0.0724827357
C,1.8752367261,3.1676165696,-0.0066353905
C,0.9650868917,2.1793132285,-0.2443961169
C,1.6872561445,0.9372857629,-0.315218853
C,7.9719462973,-0.0819339057,0.3212967441
C,1.1228921901,-0.3093773594,-0.5436588578
C,4.699950015,-3.6348971332,-0.5324617549
C,9.4566591475,-0.0339960621,0.5084961594
C,10.3220949102,0.1407331755,-0.5767071302
C,10.0350928983,-0.1590164597,1.7761559735
C,11.7055570408,0.1893384614,-0.4163828017
C,11.4150957687,-0.1140100398,1.9648443426
C,12.2521841161,0.0611969536,0.8616220983
C,4.7734382052,-5.1193415097,-0.713351209
C,4.9494563399,-5.6908797536,-1.9780816137
C,4.6658036098,-5.9916722248,0.3750542568
C,5.0173932521,-7.070733989,-2.1608971032
C,4.730289151,-7.3751537355,0.2206005541
C,4.9068997229,-7.9147774737,-1.0545977914
H,0.1453297478,-2.948417077,-1.000269924
H,2.1103703197,-4.7887123555,-0.9955459979
H,7.3815595855,-4.6162166528,-0.3315243888
H,9.1686261294,-2.6757026932,0.1348301414
H,8.909731485,2.5944970244,0.7785298984
H,6.9160896123,4.4037269631,0.7671434285
H,1.6978751856,4.2284765118,0.1075598462
H,-0.1061427857,2.2695559962,-0.3637446371
N,3.1724839924,-1.6861227145,-0.4696250702
N,5.9059628667,1.282089053,0.2440202577
N,6.0405500051,-1.5860943101,-0.1067695489
N,3.0271889333,1.1919285995,-0.1189102973
F,9.8159054445,0.2662676455,-1.814524475
F,12.5051272045,0.3570334643,-1.4719060049
F,13.5718325943,0.1061929476,1.028325602
F,11.9367989811,-0.2365281066,3.1872922348
F,9.2468672982,-0.3280971889,2.8505330887
F,5.0576927577,-4.8959130708,-3.0553412866
F,5.1861809175,-7.5857430512,-3.3806679046
F,4.9698433528,-9.2343885105,-1.2156901311
F,4.6243989315,-8.181385579,1.279062478
F,4.4952328187,-5.4923039,1.6102519319
H,0.0478678916,-0.3487547861,-0.6799648931
C,4.3671526577,3.2134648047,0.3036270018
H,4.318565867,4.2886905593,0.4354544763

Cartesian Coordination of 1au$^+$**-2ni**$^-$

–5992.5042313 hartree

C,-2.5947704478,-1.2621243134,-4.0574354473
C,-3.3130310491,-2.4182328105,-4.3823074756
C,-4.3754251643,-2.3995509662,-5.2817936363
C,-4.7365774353,-1.2031426727,-5.8972346609
C,-4.0271202259,-0.0398157433,-5.6120137877
C,-2.9699639543,-0.0860033726,-4.7110715197
C,-3.2508299966,3.9357556259,-2.0257876647
C,-4.1073553579,4.5370368367,-2.9443899019
C,-5.0559384451,3.7667741738,-3.6132160636
C,-5.1402804902,2.4041349985,-3.3485219094
C,-4.2727982548,1.829706496,-2.4269669027
F,-2.9917335241,-3.5890518143,-3.8156351245
F,-5.0430432263,-3.521950064,-5.5636067092
F,-5.7532288363,-1.1741811059,-6.7580344027
F,-4.3679387738,1.1151538839,-6.1901581561
F,-2.3190189772,1.0594483443,-4.4608512855
F,-4.0306844829,5.849942901,-3.1832692722
F,-5.8801994846,4.3327482543,-4.4969051386
F,-6.0382023986,1.6451564517,-3.9925532388
F,-4.3849951839,0.5024407688,-2.2292674646
H,-0.5082011335,-0.62411839,-5.634891981
Au,1.0308446571,-1.5285607701,-0.6992793429
C,-1.5090462256,-1.2959999993,-3.0392060171
C,-1.8550696015,-1.6677577573,-1.7386489756
C,-3.1892062902,-1.953014248,-1.27513795
C,-3.1022078588,-2.3525356741,0.0225132332
C,-1.7151922708,-2.3221975615,0.3921766474
C,-1.2183102294,-2.6915037571,1.6253649536
C,0.1038473333,-2.5951378073,2.0259043457
C,0.6014012717,-2.918710987,3.3281899529
C,1.9301661731,-2.5941794013,3.3513979429
C,2.2721732005,-2.0944001387,2.0522385002
C,3.5313223904,-1.6662381255,1.6576944159
C,3.8789093761,-1.2577579305,0.3881963415

C,5.1940899579,-0.8408963641,-0.0104513872
C,5.1476391777,-0.5415746165,-1.336480745
C,3.7975492337,-0.7594573693,-1.7854440877
C,3.334304717,-0.5439831634,-3.08355459
C,4.3416190258,-0.1591809837,-4.1083201904
C,5.3638751802,-1.0475641499,-4.4607370103
C,6.3487352369,-0.7137464102,-5.3843739373
C,6.3186906538,0.5388587353,-5.9929024072
C,5.3070815329,1.4406401501,-5.6735897244
C,4.3346225349,1.0887504452,-4.7400530921
C,2.0038340637,-0.6695854425,-3.475213764
C,1.5190899573,-0.474692411,-4.8133996307
C,0.17316818,-0.6775875073,-4.8005822515
C,-0.2079832287,-1.0044127051,-3.4536131288
C,2.5971653322,1.8870109225,-0.6636552221
C,2.8584348489,1.7035315407,0.7410692801
C,4.151001784,1.9204344363,1.2866226341
C,4.0612070121,1.6432480831,2.6281796517
C,2.7116843932,1.2423018689,2.8733893725
C,2.2303306276,0.7922985455,4.1055386868
C,3.1434409669,0.8430794027,5.2827743341
C,4.1792852791,-0.0680411814,5.4882527162
C,5.028679222,0.0042383424,6.5881056812
C,4.8469922588,1.0163377223,7.5268895122
C,3.8196399387,1.9411316099,7.3568408318
C,2.9865633908,1.8465685241,6.2447280015
C,0.9442776886,0.3278411336,4.3013883143
C,0.4927433643,-0.280438689,5.5263206292
C,-0.8006195003,-0.645655379,5.3335878011
C,-1.1656657101,-0.2141101511,4.0037040342
C,-2.4632960498,-0.2706954104,3.5034296229
C,-3.5510426837,-0.758538305,4.3952572184
C,-3.9169460023,-0.0355082121,5.5387366613
C,-4.9406738779,-0.4510498544,6.3859963322
C,-5.6499997474,-1.6131075282,6.0920972686
C,-5.3229902922,-2.3504202954,4.9577889279
C,-4.2873600383,-1.9162969371,4.1359807768
C,-2.8162520543,0.2993855885,2.2796733079
C,-4.1843685753,0.4903459951,1.863891255
C,-4.1493258138,1.1847165045,0.696896836
C,-2.7632793611,1.3748343286,0.3606623966
C,-2.3168847084,1.9513505386,-0.8111868504
C,-3.2987037069,2.5630768551,-1.7488669272
C,-0.960771066,2.0188536932,-1.1659848616
C,-0.4852478933,2.433631369,-2.4458887045
C,0.8845022429,2.3640570404,-2.4032224221
C,1.2273768767,1.9310219743,-1.0991016186
F,5.4152272013,-2.2632019811,-3.8971056229
F,7.312400785,-1.5859533708,-5.6961714608
F,7.2527375702,0.8700915727,-6.8851071837
F,5.2730829972,2.6378969639,-6.2644851658
F,3.3734167086,1.9785165824,-4.4861459105
F,4.3835021264,-1.0785980722,4.6182101213
F,6.0072311152,-0.893522774,6.7512426998
F,5.6522707496,1.096223855,8.5881784752
F,3.6439108068,2.9122812893,8.2575113002
F,2.0136780449,2.7532479924,6.1095412295
F,-3.277005098,1.0981463755,5.8456784017
F,-5.258007425,0.264149196,7.4696806152
F,-6.6368634289,-2.0187535127,6.8940749579
F,-5.995357635,-3.4705393246,4.6690892248
F,-4.0075293867,-2.6758744935,3.0547263359
F,-2.3621489746,4.7152894827,-1.4018480782
H,-4.0792252879,-1.831155606,-1.8690291622
H,-3.8987871452,-2.6227735652,0.6977851716
H,-1.931731829,-3.0479106635,2.3535505736
H,-0.0087356168,-3.302586472,4.1317425631
H,2.6301313533,-2.6673191282,4.168898218
H,4.3077801128,-1.6539628132,2.4089130181
H,6.0352482173,-0.7748950069,0.6640461407
H,5.9401746022,-0.1588949961,-1.9590053661
H,2.139848424,-0.2216828817,-5.6584736726
H,4.9969557234,2.2448583406,0.7011983115
H,4.8362484619,1.7058901107,3.3780578586
H,1.1042206,-0.4277637645,6.4043058684
H,-1.4631496756,-1.1402384877,6.0279871889
H,-5.0517196775,0.1702605731,2.4213761152
H,-4.9849791137,1.5289049189,0.1074954476
H,-1.1084886987,2.7281031933,-3.2744161276
H,1.6071765202,2.6045482237,-3.1642974394
N,-0.9824520719,-1.8847003508,-0.6946252613
N,1.1456570221,-2.1115998233,1.2628722934
N,3.0487765785,-1.1899006514,-0.7155727876
N,0.9281491582,-0.9996036037,-2.6723028548
N,1.9682489954,1.3022520398,1.7020906982
N,-0.0809460517,0.3462389381,3.3674853342
N,-1.9516525046,0.83513341,1.3560333246
N,0.1035438824,1.6967824983,-0.3380233974
Ni,0.0086781759,1.0386730681,1.520801824
O,3.5395650366,2.0511921598,-1.4707788866

[S9] M. J. Frisch, G. W. Trucks, H. B. Schlegel, G. E. Scuseria, M. A. Robb, J. R. Cheeseman, G. Scalmani, V. Barone, G. A. Petersson, H. Nakatsuji, X. Li, M. Caricato, A. V. Marenich, J. Bloino, B. G. Janesko, R. Gomperts, B. Mennucci, H. P. Hratchian, J. V. Ortiz, A. F. Izmaylov, J. L. Sonnenberg, D. Williams-Young, F. Ding, F. Lipparini, F. Egidi, J. Goings, B. Peng, A. Petrone, T. Henderson, D. Ranasinghe, V. G. Zakrzewski, J. Gao, N. Rega, G. Zheng, W. Liang, M. Hada, M. Ehara, K. Toyota, R. Fukuda, J. Hasegawa, M. Ishida, T. Nakajima, Y. Honda, O. Kitao, H. Nakai, T. Vreven, K. Throssell, J. A. Montgomery, Jr., J. E. Peralta, F. Ogliaro, M. J. Bearpark, J. J. Heyd, E. N. Brothers, K. N. Kudin, V. N. Staroverov, T. A. Keith, R. Kobayashi, J. Normand, K. Raghavachari, A. P. Rendell, J. C. Burant, S. S. Iyengar, J. Tomasi, M. Cossi, J. M. Millam, M. Klene, C. Adamo, R. Cammi, J. W. Ochterski, R. L. Martin, K. Morokuma, O. Farkas, J. B. Foresman, D. J. Fox, *Gaussian 16*, Revision C.01, Gaussian, Inc., Wallingford CT, 2016.

[S10] Z. Chen, C. S. Wannere, C. Corminboeuf, R. Puchta, P. v. R. Schleyer, *Chem. Rev.* **2005**, *105*, 3842–3888.

[S11] R. Herges, D. Geuenich, *J. Phys. Chem. A* **2001**, *105*, 3214– 3220.

[S12] M. J. S. Phipps, T. Fox, C. S. Tautermann, C.-K. Skylaris, *Chem. Soc. Rev.* **2015**, *44*, 3177–3211.

[S13] Articles for *GAMESS*: a) M. W. Schmidt, K. K. Baldridge, J. A. Boatz, S. T. Elbert, M. S. Gordon, J. H. Jensen, S. Koseki, N. Matsunaga, K. A. Nguyen, S. Su, T. L. Windus, M. Dupuis, J. A. Montgomery, *J. Comput. Chem.* **1993**, *14*, 1347–1363; b) M. S. Gordon, M. W. Schmidt, In *Theory and Applications of Computational Chemistry: the first forty years* (Eds. C. E. Dykstra, G. Frenking, K. S. Kim, G. E. Scuseria) Elsevier, **2005**, pp. 1167–1189.

[S14] Report for FMO: K. Kitaura, E. Ikeo, T. Asada, T. Nakano, M. Uebayasi, *Chem. Phys. Lett.* **1999**, *313*, 701–706.

[S15] Report for pair interaction energy decomposition analysis (PIEDA): D. G. Fedorov, K. Kitaura, *J. Comput. Chem.* **2007**, *28*, 222–237.

4. Solution-state properties

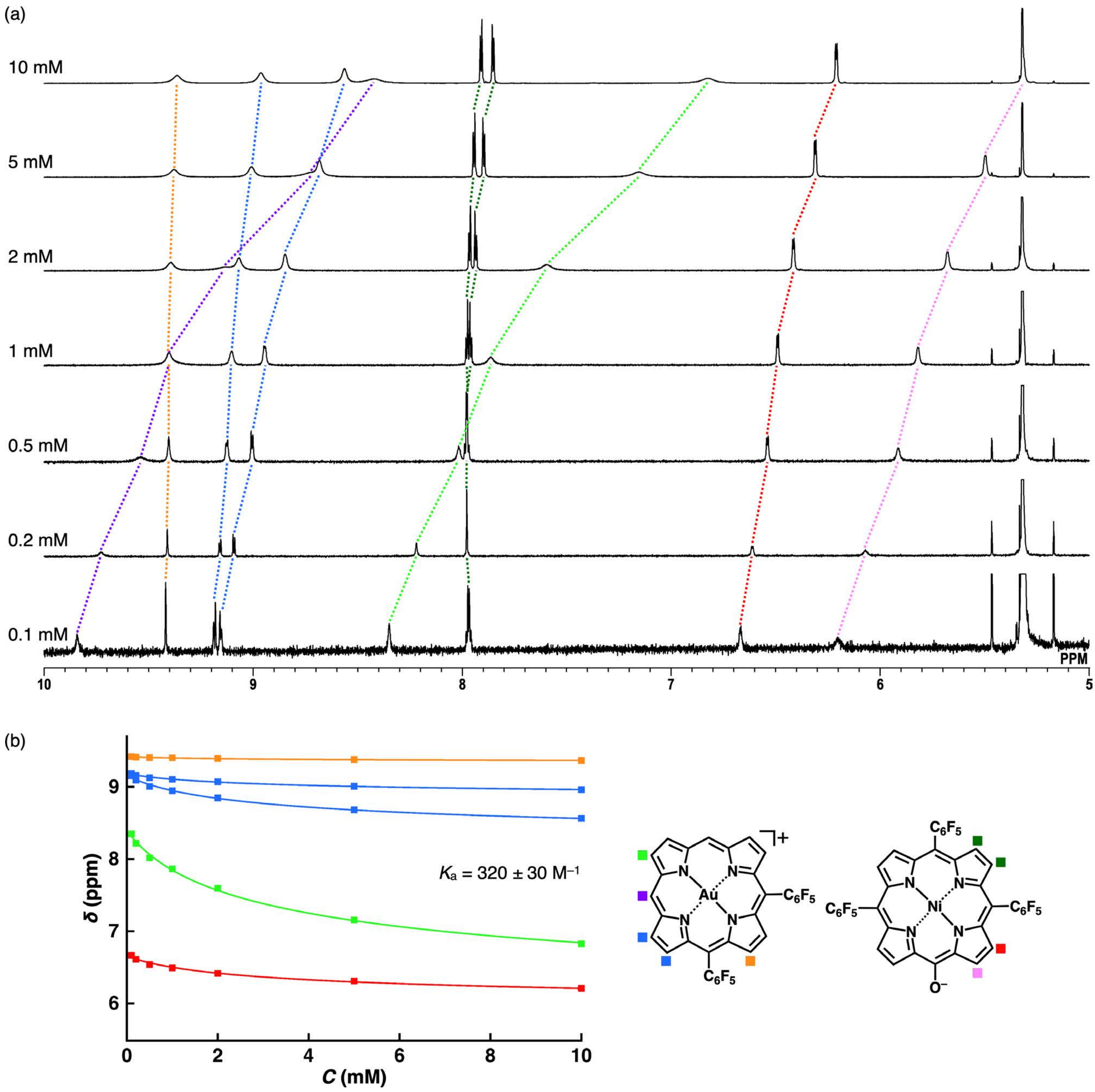

Figure S33 (a) ^{1}H NMR spectral changes at various concentrations and (b) concentration-dependent plots and fitting curves of **1au**$^+$-**2ni**$^-$ in CD_2Cl_2. Hetero-dimerization constant $K_{\rm dim}$ was estimated to be 320 M^{-1} by least-square curve fitting using the equation (1):

$$\sigma_{\rm obs} = \sigma_{\rm mono} + \frac{1+2K_{\rm dim}C-\sqrt{1+4K_{\rm dim}C}}{2K_{\rm dim}C}(\sigma_{\rm dim} - \sigma_{\rm mono}) \qquad (1)$$

for the equilibrium between the monomer and the hetero dimer showing their averaged signals ($\delta_{\rm obs}$: observed chemical shifts, $\delta_{\rm mono}$: ideal monomer chemical shift, $\delta_{\rm dim}$: ideal hetero dimer chemical shift, $K_{\rm dim}$: hetero-dimerization constant, C: total concentration of the ion pair). The signals of **1au**$^+$-**2ni**$^-$ are derived from the averaged signals between the *π-sip* and dissociated forms, the former of which would also be the averaged state between the theoretically estimated stacked structure (Figure S28) and the enantiomer. Consistently with the theoretical discussions (Figure S28), the $K_{\rm dim}$ value of **1au**$^+$-**2ni**$^-$ is smaller than that of **3au**$^+$-**2ni**$^-$ (2.8×10^5 M).[4b] In addition, the detailed assignment of **3au**$^+$ in the *π-sip* will be discussed elsewhere, whereas that of **2ni**$^-$ follows the previous report.[4b] Furthermore, examinations of the self-dimerization constant of **1au**$^+$, which should be much smaller than the $K_{\rm dim}$ value, would require the solubility for less polar solvents in the combination with appropriate counteranions.
